# Extracellular pyridine nucleotides trigger plant systemic immunity through a lectin receptor kinase/BAK1 complex

**DOI:** 10.1038/s41467-019-12781-7

**Published:** 2019-10-22

**Authors:** Chenggang Wang, Xiaoen Huang, Qi Li, Yanping Zhang, Jian-Liang Li, Zhonglin Mou

**Affiliations:** 1 Department of Microbiology and Cell Science, University of Florida, Gainesville, FL 32611 USA; 2 Department of Plant Pathology, University of Florida, Gainesville, FL 32611 USA; 3 Interdisciplinary Center for Biotechnology Research, University of Florida, Gainesville, FL 32610 USA; 4 National Institute of Environmental Health Sciences, NIH, Research Triangle Park, NC 27709 USA; 5 Present Address: Citrus Research and Education Center, University of Florida, 700 Experiment Station Road, Lake Alfred, FL 33850 USA

**Keywords:** Immunology, Plant sciences

## Abstract

Systemic acquired resistance (SAR) is a long-lasting broad-spectrum plant immunity induced by mobile signals produced in the local leaves where the initial infection occurs. Although multiple structurally unrelated signals have been proposed, the mechanisms responsible for perception of these signals in the systemic leaves are unknown. Here, we show that exogenously applied nicotinamide adenine dinucleotide (NAD^+^) moves systemically and induces systemic immunity. We demonstrate that the lectin receptor kinase (LecRK), LecRK-VI.2, is a potential receptor for extracellular NAD^+^ (eNAD^+^) and NAD^+^ phosphate (eNADP^+^) and plays a central role in biological induction of SAR. LecRK-VI.2 constitutively associates with BRASSINOSTEROID INSENSITIVE1-ASSOCIATED KINASE1 (BAK1) in vivo. Furthermore, BAK1 and its homolog BAK1-LIKE1 are required for eNAD(P)^+^ signaling and SAR, and the kinase activities of LecR-VI.2 and BAK1 are indispensable to their function in SAR. Our results indicate that eNAD^+^ is a putative mobile signal, which triggers SAR through its receptor complex LecRK-VI.2/BAK1 in *Arabidopsis thaliana*.

## Abstract (web summary)

Systemic signals allows plants to mount immune responses in sites that are distal from the local infection site. Here, the authors provide evidence that nicotinamide adenine dinucleotide (NAD ^+^ ) is a potential systemic signal that induces immunity via the lectin receptor kinase LecRK-VI.2 and BAK1.

## Introduction

All eukaryotes possess a sophisticated immune system to defend against microbial pathogens. Whereas animals employ both innate and adaptive immunities, plants lack adaptive immunity and strictly rely on innate immunity. Every plant cell is capable of using the so-called two-tiered innate immune system to detect potential microbial pathogens and mount immune responses^1^. In the local leaves where the initial infection occurs, plant cells utilize pattern recognition receptors (PRRs) to sense conserved features on/in the invading microbes, which are named pathogen-associated molecular patterns (PAMPs), and activate PAMP-triggered immunity (PTI). Successful pathogens employ effectors to dampen PAMP signaling. Resistant plants in turn exploit resistance proteins to detect the presence of pathogen effectors, inducing effector-triggered immunity. These plant–pathogen battles often cause injuries to the plant cells, resulting in release of host-derived damage-associated molecular patterns (DAMPs) that further potentiate immunity through PRRs^2^.

Pathogen perturbation in the local leaves induces production of mobile signals that are transported to the uninfected parts (the systemic leaves) of the plant, leading to the activation of a long-lasting form of immunity known as systemic acquired resistance (SAR) that confers resistance to subsequent infections by a broad spectrum of pathogens^3^. A group of structurally unrelated molecules have been proposed as SAR mobile signals, which include the lipid transfer protein Defective in Induced Resistance1 (DIR1)^4^, salicylic acid (SA) and its derivative methyl SA^5–7^, dehydroabietinal^8^, azelaic acid (AzA)^9^, glycerol-3-phosphate (G3P)^10^, pipecolic acid (Pip) and its derivative *N*-hydroxy-Pip (NHP)^11–13^, and monoterpenes^14^. In addition, nitric oxide (NO) and reactive oxygen species (ROS) have been implicated in the SAR signaling amplification loop^15,16^. It has been suggested that NO, ROS, AzA, and G3P function in a pathway in parallel with the SA pathway in SAR signaling and that Pip acts upstream of the NO-ROS-AzA-G3P branch^7,16^. However, how these SAR signals are perceived in the systemic leaves remains unclear.

Nicotinamide adenine dinucleotide (NAD^+^) and NAD^+^ phosphate (NADP^+^) are universal electron carriers functioning in both metabolic reactions and intracellular signaling^17^. Accumulating evidence has indicated that cellular NAD(P)^+^ can also be released into the extracellular space to stimulate outside-in signaling^18^. In plants, extracellular NAD(P)^+^ [eNAD(P)^+^] elicits transcriptional and metabolic changes similar to those induced by pathogen infection, and pathogen infection leads to leakage of NAD(P)^+^ into the extracellular fluid at concentrations sufficient for immune activation^19,20^. Furthermore, transgenic expression of the human NAD(P)^+^-hydrolyzing ectoenzyme CD38 in *Arabidopsis thaliana* (*Arabidopsis*) partially compromises biological induction of SAR^21^. These results suggest that eNAD(P)^+^ may be an SAR signal molecule. Recently, we identified the *Arabidopsis* legume-like lectin receptor kinase (LecRK), LecRK-I.8, as a potential eNAD^+^ receptor^22^. However, LecRK-I.8 does not bind NADP^+^ and mutations in *LecRK-I.8* have no effect on biological induction of SAR^22^. Thus, the identity of the eNADP^+^-binding receptor and whether eNAD(P)^+^ is an SAR signal molecule remain to be uncovered.

In this study, we show that eNAD^+^ is a putative SAR mobile signal and demonstrate that the eNAD(P)^+^ receptor complex LecRK-VI.2/BAK1 (Brassinosteroid insensitive1-Associated Kinase1) is a key signaling component of SAR in *Arabidopsis*.

## Results

### Exogenous NAD^+^ and/or its derivative(s) move systemically

**Fig. 1:**
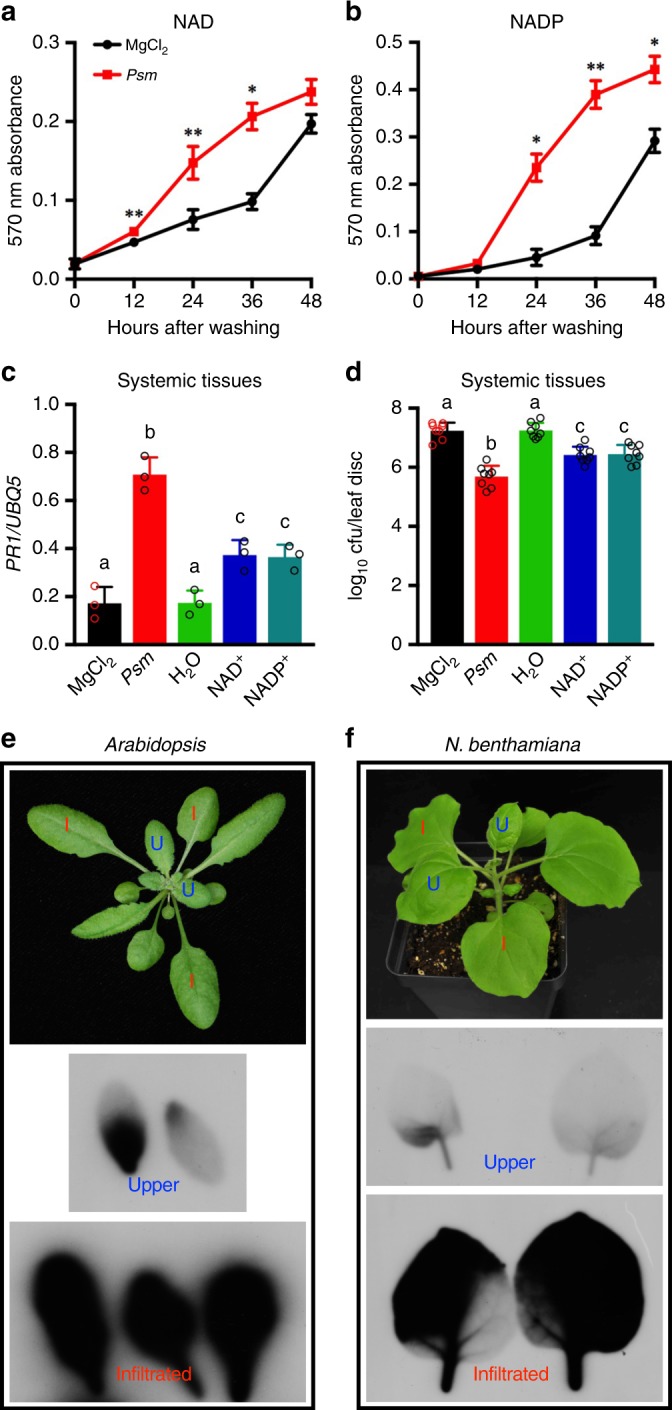
Induction of systemic resistance by exogenous NAD(P)^+^ and movement of exogenously applied NAD^+^. **a**, **b** NAD **a** and NADP **b** leakage from the wild-type Col-0 leaves infiltrated with 10 mm MgCl_2_ (mock) or *Psm* (OD_600_ = 0.002). One leaf disk was removed from each infiltrated leaf and sets of 10 leaf disks were submerged in 5 mL water in test tubes. NAD(P) concentrations in the water were measured over time by enzymatic cycling assays. Data represent the mean ± standard deviation (SD) of three biological replicates. Asterisks denote significant differences between *Psm*- and MgCl_2_-treated samples (*p < 0.05, **p < 0.01; Student’s *t* test). **c**, **d** Expression of *PR1* **c** and growth of *Psm* **d** in the systemic leaves of the wild-type Col-0 plants treated with MgCl_2_, *Psm*, H_2_O, NAD^+^, or NADP^+^. For MgCl_2_ and *Psm* treatments, three lower leaves on each 4-week-old soil-grown *Arabidopsis* plant were infiltrated with 10 mm MgCl_2_ or a *Psm* suspension (OD_600_ = 0.002). Two days later, two systemic leaves were either collected for *PR1* expression analysis by qPCR **c** or challenge-inoculated with *Psm* (OD_600_ = 0.001) **d**. Three days later, eight leaves were collected to examine the growth of the pathogen. Alternatively, three lower leaves were infiltrated with H_2_O, 0.4 mm NAD^+^, or 0.8 mm NADP^+^ every 12 hr for a total of four times. About 5 hr after the last infiltration, two systemic leaves were either collected for *PR1* analysis **c** or challenge-inoculated with *Psm* (OD_600_ = 0.001) **d**. Expression levels of *PR1* **c** were normalized against the constitutively expressed *UBQ5*. Data represent the mean ± SD of three **c** or eight biological replicates **d**. Different letters denote significant differences (*p* < 0.05; one-way ANOVA with Tukey’s test). Compared with the strong systemic resistance induced by *Psm* (~ 35-fold decrease in *Psm* growth), NAD^+^ and NADP^+^ induced intermediate levels of resistance in the systemic leaves (~ 6.5-fold decrease in *Psm* growth). **e**, **f** Autoradiographic detection of ^32^P in the systemic leaves of *Arabidopsis* **e** and *N. benthamiana* **f** plants with the lower leaves infiltrated with ^32^P-NAD^+^. Three lower leaves on an *Arabidopsis* plant or two lower leaves on a *N. benthamiana* plant were infiltrated with a water solution of 6.25 nm ^32^P-NAD^+^ plus 1 mm unlabeled NAD^+^. Twenty-four hr later, the infiltrated leaves (I in red) and two systemic leaves (U in blue) were collected and exposed to X-ray film

We have shown that exogenous application of NAD(P)^+^ induces immune responses and that depletion of eNAD(P)^+^ by transgenic expression of the human NAD(P)^+^-hydrolyzing ectoenzyme CD38 partially compromises biological induction of SAR in *Arabidopsis*^19,21^. These results strongly suggest that eNAD(P)^+^ may be an SAR signal molecule. However, when infiltrated into lower leaves of *Arabidopsis* plants, only NAD^+^ at a concentration (5 mm) higher than physiological levels (~0.4 mm) was able to induce a partial and significant resistance (intermediate resistance) in the systemic leaves^21^. We reasoned that, during pathogen infection, NAD(P)^+^ might continuously leak into the extracellular space to trigger SAR. To test this hypothesis, we measured the virulent bacterial pathogen *Pseudomonas syringae* pv. *maculicola* ES4326 (*Psm*)-induced NAD(P) (both oxidized and reduced forms) leakage from leaf disks floating on water. As shown in Fig. 1a, b, the amounts of NAD(P) in the water surrounding the *Psm*-infiltrated leaf disks were significantly higher than those surrounding the mock (10 mm MgCl_2_)-infiltrated leaf disks in the first 36 h. To test whether NAD(P) could leak out of intact leaves, detached leaves were submerged in water with the petioles above the water surface. As shown in Supplementary Fig. 1a, b, NAD(P) levels in the water covering *Psm*-infiltrated leaves increased significantly at 36 and 48 hour. The slower kinetics of NAD(P) leakage from intact leaves than from leaf disks may be attributed to the process of diffusing out of the apoplast. Nevertheless, these results indicate that significant amounts of NAD(P) leaked into the extracellular space after *Psm* infection.

To mimic the eNAD(P)^+^ dynamics during pathogen infection, we infiltrated three lower leaves on each *Arabidopsis* plant with 0.4 mm NAD^+^ or 0.8 mm NADP^+^ every 12 hours for a total of four times. Approximately 5 hours after the last infiltration, the systemic leaves were collected for analysis of the induction of *Pathogenesis-Related gene1* (*PR1*), a marker gene of SAR^23^. To test NAD(P)^+^-induced systemic resistance, the systemic leaves were challenge-inoculated with *Psm* and the in planta bacterial growth was determined 3 days later. Meanwhile, *Psm*-triggered biological induction of SAR was used as the positive control. To this end, three lower leaves on each plant were infiltrated with *Psm*. Two days later, the systemic leaves were either collected for *PR1* analysis or challenge-inoculated with *Psm* for resistance test. As shown in Fig. 1c, d, treatment of lower leaves with NAD(P)^+^ significantly induced expression of *PR1* and resistance to *Psm* in the systemic leaves, though the induction levels were significantly lower than those triggered by *Psm*, which is consistent with the notion that multiple signal molecules are involved in SAR activation^24^. Taken together, these results suggest that eNAD(P)^+^ may function as an SAR signal molecule in *Arabidopsis*.

NAD(P)^+^ may act in the infiltrated (local) leaves or be transported to the systemic leaves to trigger resistance. To test whether eNAD(P)^+^-triggered systemic resistance is associated with translocation of eNAD(P)^+^ from local to systemic leaves, an NAD^+^ solution (1 mm unlabeled NAD^+^ plus 6.25 nm ^32^P-NAD^+^) was infiltrated into lower leaves on *Arabidopsis* and *Nicotiana benthamiana* plants (Fig. 1e, f). Twenty-four hours later, the systemic leaves were collected for autoradiographic detection of ^32^P. As shown in Fig. 1e, f, infiltration of ^32^P-NAD^+^ resulted in movement of the radioactivity into the systemic leaves in both *Arabidopsis* and *N. benthamiana*. To define the portion of radioactivity that moves systemically, *Arabidopsis* leaves were infiltrated with 6.25 nm ^32^P-NAD^+^ (without unlabeled NAD^+^) and the radioactivity in the local and systemic leaves was quantified. As shown in Supplementary Fig. 2a, ~ 10.8% and 6.1% of the radioactivity moved into the systemic leaves in the wild type and the *35* *S:CD38* transgenic plants^21^, respectively. Furthermore, whether NAD^+^ per se moves systemically was tested by paper chromatography. As shown in Supplementary Fig. 2b, although three bands were detected in the extracts of local and systemic leaves, the predominant band has the same size as ^32^P-NAD^+^. This result supports the hypothesis that eNAD^+^ can be transported from local to systemic leaves. Interestingly, the signal intensity of the potential ^32^P-NAD^+^ band in the extract of *35* *S:CD38* systemic leaves was significantly reduced (Supplementary Fig. 2b), indicating that the movement of ^32^P-NAD^+^ in the *35* *S:CD38* plants was inhibited. This observation is line with the previously reported result that SAR induction is partially compromised in *35* *S:CD38* plants^21^.

### LecRK-VI.2 is a potential receptor for eNAD(P^)+^

**Fig. 2:**
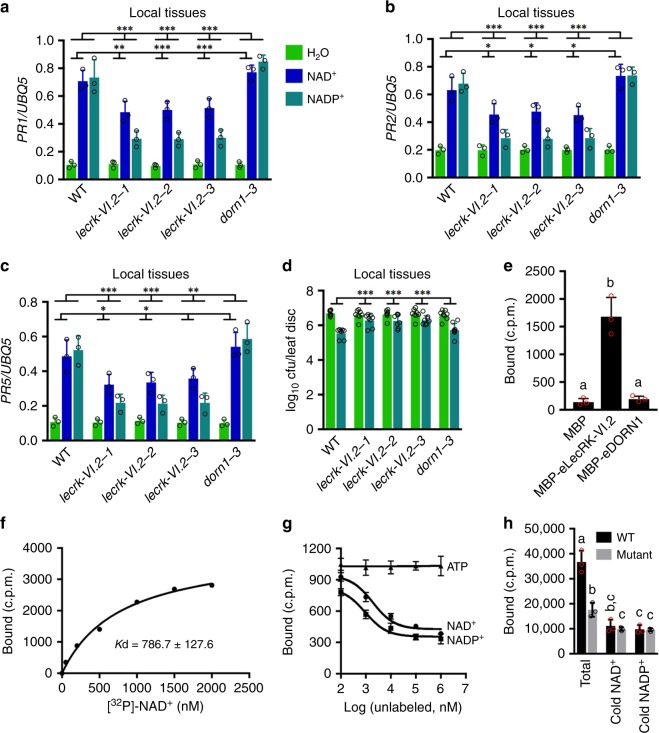
Exogenous NAD(P)^+^-induced local defense responses in the *lecrk-VI.2* mutants and binding of NAD(P)^+^ to LecRK-VI.2. **a**–**c** Exogenous NAD(P)^+^-induced local expression of *PR1* **a**, *PR2* **b**, and *PR5* **c** in the wild-type (WT), *lecrk-VI.2*, and *dorn1-3* plants. Leaves of 4-week-old soil-grown plants were infiltrated with 0.2 mm NAD^+^, 0.4 mm NADP^+^ or H_2_O. The infiltrated leaves were collected 20 hr later. Total RNA was extracted and subjected to qPCR analysis. Expression levels were normalized against the constitutively expressed *UBQ5*. Data represent the mean ± SD of three biological replicates. Asterisks denote significant differences between the induction in the mutants and that in the wild type (*p < 0.05, **p < 0.01, ***p < 0.001; two-way ANOVA with Sidak’s test). Upper line: induction by NADP^+^; lower line: induction by NAD^+^. **d** Exogenous NADP^+^-induced local resistance in the wild-type, *lecrk-VI.2*, and *dorn1-3* plants. Leaves of 4-week-old soil-grown plants were infiltrated with 0.4 mm NADP^+^ or H_2_O. Five hr later, the infiltrated leaves were inoculated with a *Psm* suspension (OD_600_ = 0.001). Three d later, eight leaves were collected to examine the growth of the pathogen. Data represent the mean ± SD of eight biological replicates. Asterisks denote significant differences between the induction in the mutants and that in the wild type (***p < 0.001; two-way ANOVA with Sidak’s test). **e** Binding of ^32^P-NAD^+^ to recombinant MBP, MBP-eLecRK-VI.2, and MBP-eDORN1 proteins. Approximately 5 μg recombinant proteins were used for each binding assay. Data represent the mean ± SD of three experiments. Different letters denote significant differences (*p* < 0.05; one-way ANOVA with Tukey’s test). **f** Saturation binding assay for LecRK-VI.2. Recombinant MBP-eLecRK-VI.2 proteins were incubated with the indicated concentrations of ^32^P-NAD^+^ for 30 min. Free NAD^+^ was removed by washing. Data were plotted as a specific binding. The dissociation constant (*K*d) was calculated by one site specific binding saturation model using GraphPad Prism 7 (www.graphgpad.com). The experiment was repeated four times with similar results and results from a representative experiment were presented. **g** Competitive binding assay for LecRK-VI.2. Samples containing 250 nm of ^32^P-NAD^+^ in the presence of 100 nm to 1 mm of unlabeled nucleotides were assayed for specific binding of ^32^P-NAD^+^. Data were plotted as a specific binding with SD of three experiments. The 50% inhibition concentration (IC_50_) values were calculated in GraphPad Prism 7 using the one site Fit logIC_50_ competition model. **h** Binding of ^32^P-NAD^+^ to the microsomal fractions of the wild-type (WT) and *lecrk-VI.2-2* mutant plants. Samples containing 250 nm ^32^P-NAD^+^ in the absence (total) or presence of 1000-fold unlabeled (cold) NAD^+^ or NADP^+^ were assayed for binding of ^32^P-NAD^+^. Data represent the mean ± SD of three experiments. Different letters denote significant differences (*p* < 0.05; one-way ANOVA with Tukey’s test)

To convincingly demonstrate that eNAD(P)^+^ is an SAR signal molecule, it is crucial to identify its receptors and test their role in SAR responses. We have previously identified LecRK-I.8 as a potential eNAD^+^-binding receptor in *Arabidopsis*^22^. LecRK-I.8 binds NAD^+^ with a dissociation constant (K*d*) of 436.5 ± 104.8 nm. Surprisingly, NADP^+^ did not compete for binding of LecRK-I.8 with ^32^P-NAD^+^. In line with this result, mutations in *LecRK-I.8* did not affect NADP^+^-induced immune responses. Furthermore, the *lecrk-I.8* mutations compromised basal immunity but had no effect on biological induction of SAR^22^. These results prompted us to identify eNADP^+^-binding receptors. We have previously isolated transferred DNA (T-DNA) insertion lines for a group of receptor-like genes that were induced by exogenous NAD^+^^22^. We suspected that NAD^+^ might also induce eNADP^+^-binding receptor genes and thus tested exogenous NADP^+^-induced *Psm* resistance in the T-DNA insertion lines. As shown in Supplementary Fig. 3a, NADP^+^-induced resistance was clearly reduced in the T-DNA insertion line SALK_070801, which harbors a T-DNA insertion in the gene At5g01540 that encodes LecRK-VI.2. We then tested the transcript levels of *LecRK-VI.2* in SALK_070801 and two more T-DNA insertion lines, SAIL_1146_B02 and SAIL_796_E11. Basal transcript levels of *LecRK-VI.2* in the T-DNA insertion lines were ~ 33–48% of those in the wild type, indicating that all three T-DNA insertion lines are knockdown mutants of *LecRK-VI.2* (Supplementary Fig. 3b, c). SALK_070801 has previously been named *lecrk-VI.2-1*^25^, and we thus named SAIL_1146_B02 and SAIL_796_E11 *lecrk-VI.2-2* and *lecrk-VI.2-3*, respectively (Supplementary Fig. 3b). Although previous work has shown that NAD^+^-induced resistance to *Psm* was not affected in the three *lecrk-VI.2* alleles^22^, NAD^+^-induced expression of *PR1*, *PR2*, and *PR5* were all significantly inhibited with the exception of *PR5* in *lecrk-VI.2-3* (Fig. 2a–c). Importantly, NADP^+^-induced *PR* gene expression and *Psm* resistance were significantly suppressed in all three alleles but not in the extracellular ATP receptor mutant *dorn1-3*^26^ (Fig. 2a–d). These results suggest that LecRK-VI.2 may play a more important role in eNADP^+^ signaling than in eNAD^+^ signaling in the local leaves.

We then tested whether LecRK-VI.2 binds NAD^+^ using ^32^P-NAD^+^. The extracellular domain (amino acids (AAs) 23-310) of LecRK-VI.2 (eLecRK-VI.2) was expressed and purified from *Escherichia coli* as a recombinant maltose-binding protein (MBP) fusion (Supplementary Fig. 3d) and subjected to binding assays with ^32^P-NAD^+^. As shown in Fig. 2e, a specific NAD^+^ binding activity was detected for MBP-eLecRK-VI.2 but not for the simultaneously purified MBP and MBP-eDORN1 (AA 22–288) proteins. MBP-eLecRK-VI.2 exhibited a typical saturation curve for NAD^+^ binding with a K*d* of 786.7 ± 127.6 nm (Fig. 2f). To test whether LecRK-VI.2 binds NADP^+^, we compared the abilities of unlabeled NAD^+^, NADP^+^, and ATP to compete for binding of ^32^p-NAD^+^. Binding of ^32^P-NAD^+^ to MBP-eLecRK-VI.2 was effectively competed by unlabeled NAD^+^ (50% inhibition concentration, IC_50_, 1,887 nm) and NADP^+^ (IC_50_, 945 nm), but was not competed by ATP (IC_50_ > 100,000 nm) (Fig. 2g). As the IC_50_ of NADP^+^ is approximately half of that of NAD^+^, the relative binding affinity of NADP^+^ is slightly higher than that of NAD^+^, which is in agreement with the compromised NADP^+^ responsiveness in the *lecrk-VI.2* mutants (Fig. 2a–d). Furthermore, the total NAD^+-^binding activity in the membrane fractions of the *lecrk-VI.2-2* mutant was significantly lower than that in the wild type, and the binding of ^32^P-NAD^+^ to the membrane fractions was also effectively competed by both unlabeled NAD^+^ and NADP^+^ (Fig. 2h). Note that the reduced binding of ^32^P-NAD^+^ to the membrane fractions of *lecrk-VI.2-2* was further competed by unlabeled NAD^+^ and NADP^+^, indicating the existence of other NAD(P)^+^-binding proteins. Taken together, these results indicate that LecRK-VI.2 is a potential receptor for both NAD^+^ and NADP^+^. In agreement with this conclusion, the *LecRK-VI.2* gene is induced by both NAD^+^ and NADP^+^ (Supplementary Fig. 3e).

### LecRK-VI.2 is required for NAD(P)^+^-induced systemic immunity

**Fig. 3:**
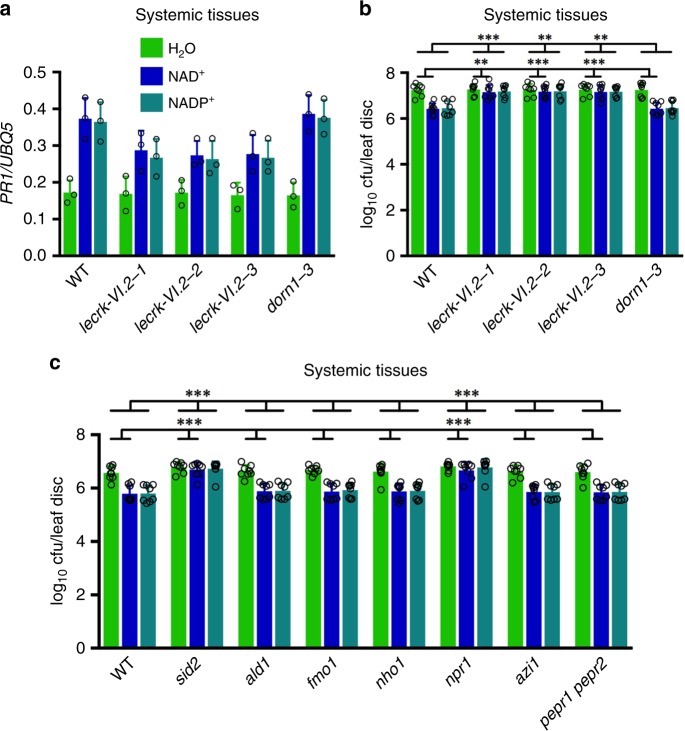
Exogenous NAD(P)^+^-induced systemic responses in *lecrk-VI.2* and several SAR mutants. **a**, **b** NAD(P)^+^-induced systemic *PR1* expression **a** and systemic resistance **b** in the wild-type (WT), *lecrk-VI.2*, and *dorn1-3* plants. Three lower leaves on each 4-week-old soil-grown plant were infiltrated with H_2_O, 0.4 mm NAD^+^, or 0.8 mm NADP^+^ every 12 hr for a total of four times. About 5 hr after the last infiltration, two systemic leaves were either collected for *PR1* analysis **a** or challenge-inoculated with a *Psm* suspension (OD_600_ = 0.001) **b**. Three d later, eight leaves were collected to examine the growth of the pathogen. Expression levels of *PR1* **a** were normalized against the constitutively expressed *UBQ5*. Data represent the mean ± SD of three **a** or eight biological replicates **b**. Asterisks denote significant differences between the induction in the mutants and that in the wild type (**p < 0.01, ***p < 0.001; two-way ANOVA with Sidak’s test). Upper line: induction by NADP^+^; lower line: induction by NAD^+^. **c** NAD(P)^+^-induced systemic resistance in the wild-type, *sid2*, *ald1*, *fmo1*, *nho1*, *npr1*, *azi1*, and *pepr1 pepr2* plants. The experiment was conducted as in **b**. Data represent the mean ± SD of eight biological replicates. Asterisks denote significant differences between the induction in the mutants and that in the wild type (***p < 0.001; two-way ANOVA with Sidak’s test). Upper line: induction by NADP^+^; lower line: induction by NAD^+^

To test whether LecRK-VI.2 is essential for exogenous NAD(P)^+^ to trigger systemic resistance, we infiltrated three lower leaves on each *Arabidopsis* plant with 0.4 mm NAD^+^ or 0.8 mm NADP^+^ every 12 hours for a total of four times. About 5 hours after the last infiltration, the systemic leaves were either collected for analysis of the induction of *PR1* or challenge-inoculated with *Psm* to test NAD(P)^+^-induced systemic resistance. As shown Fig. 3a, b, although mutations in *LecRK-VI.2* did not significantly inhibit NAD(P)^+^-induced *PR1* gene expression, NAD(P)^+^-induced resistance to *Psm* in the systemic leaves was completely blocked in the mutants plants. This result indicates that LecRK-VI.2 is required for exogenous NAD(P)^+^-induced systemic resistance and further supports LecRK-VI.2 being an eNAD(P)^+^ receptor.

To test the necessity of the well-documented SAR pathway genes in eNAD(P)^+^ signaling, we first monitored exogenous NAD(P)^+^-induced resistance to *Psm* in the local leaves of various SAR mutants. Interestingly, except *fad7* (*fatty acid desaturase7*)^27^, only mutations affecting SA accumulation, including *sid2* (*SA induction deficient2*)^28^, *eds5* (*enhanced disease susceptibility5*)^29^, *eds1*^30^, *pad4* (*phytoalexin deficient4*)^31^, and *ndr1* (*non race-specific disease resistance1*)^32^, significantly inhibited NAD(P)^+^-induced *Psm* resistance (Supplementary Fig. 4a–d). The *npr1-3* (*nonexpressor of PR genes1-3*) mutation, which largely blocks SA signaling^33^, significantly suppressed NADP^+^- but not NAD^+^-induced resistance (Supplementary Fig. 4a), which is consistent with the result obtained with the *npr1-1* allele^19^. These results indicate that eNAD(P)^+^ activates SA-dependent signaling and may function downstream or independently of *ALD1* (*AGD2-Like Defense response protein1*)^34^, *FMO1* (*Flavin-dependent Monooxygenase1*)^35^, *AZI1* (*AZelaic acid Induced1*)^9^, *NHO1* (*NONHOST1*)^36^, *PEPR1* (*PEPp Receptor1*)/*2*^37^, *DIR1*, and *SFD1* (*Suppressor of Fatty acid desaturase Deficiency1*)^38^ in the local leaves.

We further tested exogenous NAD(P)^+^-induced systemic resistance to *Psm* in the systemic leaves of *sid2*, *ald1*^34^, *fmo1*^35^, and *nho1*^36^, which do not accumulate SA, the putative SAR mobile signals Pip, NHP, and G3P, respectively, and *npr1*, *azi1*^9^, and *pepr1 pepr2*^39^, which do not respond to SA, the putative SAR signal molecule AzA, and Pep peptide elicitors, respectively. To this end, three lower leaves on each plant were infiltrated with 0.4 mm NAD^+^ or 0.8 mm NADP^+^ every 12 hours for a total of four times. Five hours after the last infiltration, the systemic leaves were challenge-inoculated with *Psm* to test NAD(P)^+^-induced systemic resistance. As shown in Fig. 3c, NAD(P)^+^-induced systemic resistance against *Psm* was significantly suppressed in *sid2* and *npr1*, but not in *ald1*, *fmo1*, *nho1*, *azi1*, and *pepr1 pepr2*, indicating that eNAD(P)^+^-triggered systemic signaling requires SA and NPR1, but not Pip, NHP, G3P, AzA, and Pep peptide elicitors.

We have previously shown that high concentrations (> 1 mm) of NAD(P)^+^ significantly induce SA accumulation^19^. To test whether the concentrations used above could induce SA accumulation, free SA levels in leaf tissues treated with 0.4 mm NAD^+^ or 0.8 mm NADP^+^ were measured. As shown in Supplementary Fig. 5a, 0.4 mm NAD^+^ and 0.8 mm NADP^+^ did not significantly induce free SA accumulation in the treated leaves, suggesting that basal SA is both necessary and sufficient for eNAD(P)^+^-induced defense signaling. On the other hand, previous microarray analysis indicated that SA treatment quickly (1 hour) induces *LecRK-VI.2* expression^40^. We confirmed this result by quantitative PCR (qPCR) and further revealed that the induction depends on the transcription coactivator NPR1 (Supplementary Fig. 5b, c). These results indicate that SA signaling positively regulates *LecRK-VI.2* expression, suggesting a signaling amplification loop comprising SA, NPR1, and LecRK-VI.2.

### LecRK-VI.2 is required for biological induction of SAR

**Fig. 4:**
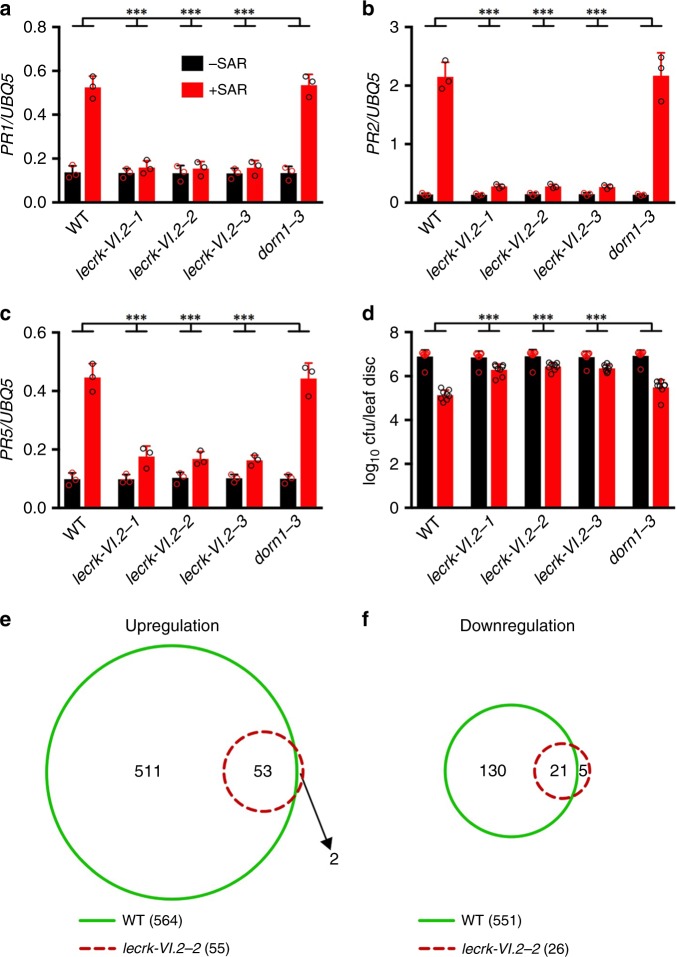
Biological induction of SAR in the *lecrk-VI.2* mutants. **a**–**c** Expression of *PR1* **a**, *PR2* **b**, and *PR5* **c** in the systemic leaves of the wild-type (WT), *lecrk-VI.2*, and *dorn1-3* plants with or without SAR induction. Three lower leaves on each 4-week-old soil-grown plant were infiltrated with 10 mm MgCl_2_ or a *Psm* suspension (OD_600_ = 0.002). Two d later, systemic leaves were collected for *PR* gene expression analysis by qPCR. Expression levels were normalized against the constitutively expressed *UBQ5*. Data represent the mean ± SD of three biological replicates. Asterisks denote significant differences between the induction in the mutants and that in the wild type (***p < 0.001; two-way ANOVA with Sidak’s test). **d** Biological induction of SAR in the wild-type, *lecrk-VI.2*, and *dorn1-3* plants. Three lower leaves on each 4-week-old soil-grown plant were infiltrated with 10 mm MgCl_2_ or a *Psm* suspension (OD_600_ = 0.002). Two d later, two systemic leaves were challenge-inoculated with *Psm* (OD_600_ = 0.001). Three d later, eight leaves were collected to examine the growth of the pathogen. Data represent the mean ± SD of eight biological replicates. Asterisks denote significant differences between the induction in the mutants and that in the wild type (***p < 0.001; two-way ANOVA with Sidak’s test). **e**, **f** Overlaps between the genes that were up- **e** or downregulated **f** in the systemic leaves of the wild-type and *lecrk-VI.2-2* plants. Plants were treated as in **a**–**c**. Total RNA extracted from the systemic tissues was subjected to microarray analysis. Genes with an absolute fold change ≥ 2 and a *q* value ≤ 0.05 were compared with obtain overlapped genes among the wild-type and *lecrk-VI.2-2* plants

If eNAD(P)^+^ is an SAR signal molecule and LecRK-VI.2 is a receptor of eNAD(P)^+^, mutations in *LecRK-VI.2* should block biological induction of SAR. To test this hypothesis, we infiltrated three lower leaves on each plant with either 10 mm MgCl_2_ (−SAR) or *Psm* (+ SAR). Two days later, expression of *PR1*, *PR2*, and *PR5* in the systemic leaves was analyzed. As shown in Fig. 4a–c, induction of the three commonly used SAR marker genes was significantly suppressed in the *lecrk-VI.2* mutants, but not in *dorn1-3*. To test SAR activation-induced resistance in the *lecrk-VI.2* plants, we challenge-inoculated the systemic leaves with *Psm* and determined the bacterial growth after 3 days. As shown in Fig. 4d, SAR activation induced strong resistance in the wild-type and *dorn1-3* plants, whereas the resistance was significantly inhibited in the *lecrk-VI.2* mutants. We further conducted a microarray experiment to compare SAR activation-induced transcriptome changes in *lecrk-VI.2-2* and wild type (National Center for Biotechnology Information Gene Expression Omnibus series number GSE121886). Systemic leaf tissues were collected two days after inoculation of the lower leaves with *Psm*, as significant systemic increases in *PR* gene expression and resistance were observed at this time point^41^ (Fig. 4a–d). Genes that were induced or suppressed twofold or higher with a low *q* value (≤0.05) by SAR activation in *lecrk-VI.2-2* and the wild type were identified and compared. As shown in Fig. 4e, f, a total of 564 and 151 genes in the wild type were up- and downregulated, respectively, whereas only 55 and 26 genes in *lecrk-VI.2-2* were up- and downregulated, respectively. In other words, transcript levels of >90 and 82% of the genes that were induced and suppressed, respectively, in the wild type were not significantly changed in the *lecrk-VI.2-2* mutant. These results indicate that the eNAD(P)^+^-binding receptor LecRK-VI.2 is a crucial component in the SAR signaling pathway.

### BAK1 and BKK1 are required for eNAD(P)^+^ signaling

**Fig. 5:**
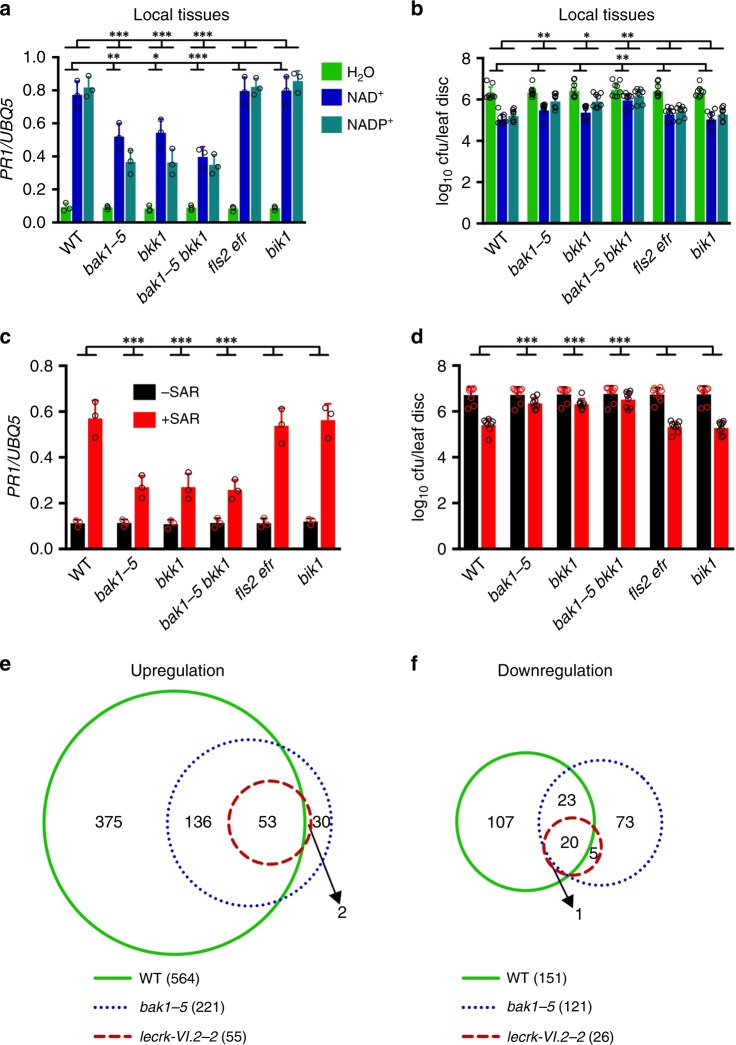
Exogenous NAD(P)^+^-induced local immune responses and biological induction of SAR in several PTI mutants. **a** Exogenous NAD(P)^+^-induced local *PR1* expression in the wild-type (WT), *bak1-5*, *bkk1*, *bak1-5 bkk1*, *fls2 efr*, and *bik1* plants. Leaves of 4-week-old soil-grown plants were infiltrated with 0.2 mm NAD^+^, 0.4 mm NADP^+^ or H_2_O. The infiltrated leaves were collected 20 hr later. Total RNA was extracted and subjected to qPCR analysis. Expression levels of *PR1* were normalized against the constitutively expressed *UBQ5*. Data represent the mean ± SD of three biological replicates. Asterisks denote significant differences between the induction in the mutants and that in the wild type (*p < 0.05, **p < 0.01, ***p < 0.001; two-way ANOVA with Sidak’s test). Upper line: induction by NADP^+^; lower line: induction by NAD^+^. **b** Exogenous NAD(P)^+^-induced local resistance in the wild-type, *bak1-5*, *bkk1*, *bak1-5 bkk1*, *fls2 efr*, and *bik1* plants. Leaves of 4-week-old soil-grown plants were infiltrated with 0.2 mm NAD^+^, 0.4 mm NADP^+^ or H_2_O. Five hr later, the infiltrated leaves were inoculated with a *Psm* suspension (OD_600_ = 0.001). Three d later, eight leaves were collected to examine the growth of the pathogen. Data represent the mean ± SD of eight biological replicates. Asterisks denote significant differences between the induction in the mutants and that in the wild type (*p < 0.05, **p < 0.01; two-way ANOVA with Sidak’s test). Upper line: induction by NADP^+^; lower line: induction by NAD^+^. **c** Expression of *PR1* in the systemic leaves of the wild-type, *bak1-5*, *bkk1*, *bak1-5 bkk1*, *fls2 efr*, and *bik1* plants with or without SAR induction. Three lower leaves on each 4-week-old soil-grown plant were infiltrated with 10 mm MgCl_2_ or a *Psm* suspension (OD_600_ = 0.002). Two d later, systemic leaves were collected for *PR1* expression analysis by qPCR. Data represent the mean ± SD of three biological replicates. Asterisks denote significant differences between the induction in the mutants and that in the wild type (***p < 0.001; two-way ANOVA with Sidak’s test). **d** Biological induction of SAR in the wild-type, *bak1-5*, *bkk1*, *bak1-5 bkk1*, *fls2 efr*, and *bik1* plants. Three lower leaves on each 4-week-old soil-grown plant were infiltrated with 10 mm MgCl_2_ or a *Psm* suspension (OD_600_ = 0.002). Two d later, two systemic leaves were challenge-inoculated with *Psm* (OD_600_ = 0.001). Three d later, eight leaves were collected to examine the growth of the pathogen. Data represent the mean ± SD of eight biological replicates. Asterisks denote significant differences between the induction in the mutants and that in the wild type (***p < 0.001; two-way ANOVA with Sidak’s test). **e**, **f** Overlaps among the genes that were up- or downregulated **f** in the systemic leaves of the wild-type, *bak1-5*, and *lecrk-VI.2-2* plants. Plants were treated as in **c**. Total RNA extracted from the systemic tissues was subjected to microarray analysis. Genes with an absolute fold change ≥ 2 and a *q* value ≤ 0.05 were compared with obtain overlapped genes among the wild-type, *bak1-5*, and *lecrk-VI.2-2* plants

It has previously been shown that LecRK-VI.2 has a positive role in PTI via constitutive association with the leucine-rich repeat receptor kinase (LRR-RK) PRR FLAGELLIN-SENSING2 (FLS2)^25,42^. Upon flagellin perception, FLS2 recruits its co-receptors BAK1 and BAK1-LIKE1 (BKK1), which phosphorylate their interacting receptor-like cytoplasmic kinase Botrytis-Induced Kinase1 (BIK1) to initiate the PTI signaling^43–48^. To test whether these well-established PTI signaling components function in eNAD(P)^+^ signaling, we tested exogenous NAD(P)^+^-induced immune responses in the *bak1-5*^49^, *bkk1*^50^, *bak1-5 bkk1*^49^, *fls2 efr* (*elongation factor Tu receptor*)^51,52^, and *bik1*^45^ mutants. As shown in Fig. 5a, b and Supplementary Fig. 6a, b, NAD(P)^+^-induced *PR* gene expression and *Psm* resistance were not significantly altered in the *fls2 efr* and *bik1* mutants, whereas the induction was differentially affected by the *bak1-5* and *bkk1* mutations. NAD^+^-induced expression of *PR1* and *PR2* was significantly inhibited in *bak1-5*, *bkk1*, and *bak1-5 bkk1*, but NAD^+^-induced expression of *PR5* and resistance to *Psm* was only significantly repressed in the *bak1-5 bkk1* double mutant. On the other hand, NADP^+^-induced *PR* gene expression and *Psm* resistance were significantly suppressed in both single and double mutants. These results indicate that BAK1 and BKK1 play overlapping and independent roles in the eNAD(P)^+^ signaling pathways.

### BAK1 and BKK1 are required for biological induction of SAR

Since BAK1 and BKK1 function in eNADP^+^ signaling, they may be required for SAR. To test this hypothesis, three lower leaves on each plant were infiltrated with either 10 mm MgCl_2_ (−SAR) or *Psm* (+SAR). Two days later, expression of *PR1*, *PR2*, and *PR5* in the systemic leaves was analyzed. Induction of the three *PR* genes was significantly reduced in the *bak1-5*, *bkk1*, and *bak1-5 bkk1* mutants (Fig. 5c and Supplementary Fig. 6c, d). We also challenge-inoculated the systemic leaves with *Psm* and determined in planta bacterial titers after 3 days. As shown in Fig. 5d, SAR activation induced strong resistance in the wild-type, *fls2 efr*, and *bik1* plants, whereas the resistance was significantly compromised in the *bak1-5*, *bkk1*, and *bak1-5 bkk1* mutants. We have included the *bak1-5* mutant in the microarray experiment described above (Fig. 4e, f) to compare SAR activation-induced transcriptome changes in *bak1-5* with those in *lecrk-VI.2-2* and the wild type. Genes that were induced or suppressed twofold or higher with a low *q* value (≤0.05) by SAR activation in *bak1-5* were identified and compared with those in the wild type and *lecrk-VI.2-2*. As shown in Fig. 5e, f, a total of 221 and 121 genes were up- and downegulated, respectively, in *bak1-5*, which fall between the numbers of genes that were up- and downregulated in the wild type and *lecrk-VI.2-2*. Furthermore, the majority of the genes that were induced or suppressed twofold or higher with a low *q* value (≤0.05) in the wild type were induced or suppressed to a smaller extent in *bak1-5* and the smallest extent in *lecrk-VI.2-2* (Supplementary Fig. 7 and Supplementary Data 1). These results indicate that the *bak1-5* mutation suppressed SAR activation-induced signaling, but the strength of the suppression was not as strong as that in *lecrk-VI.2-2*, which may be attributed to the presence of BKK1 that might have some redundant functions with BAK1 in biological induction of SAR (Fig. 5d).

### LecRK-VI.2 and BAK1 form a complex in vivo

**Fig. 6:**
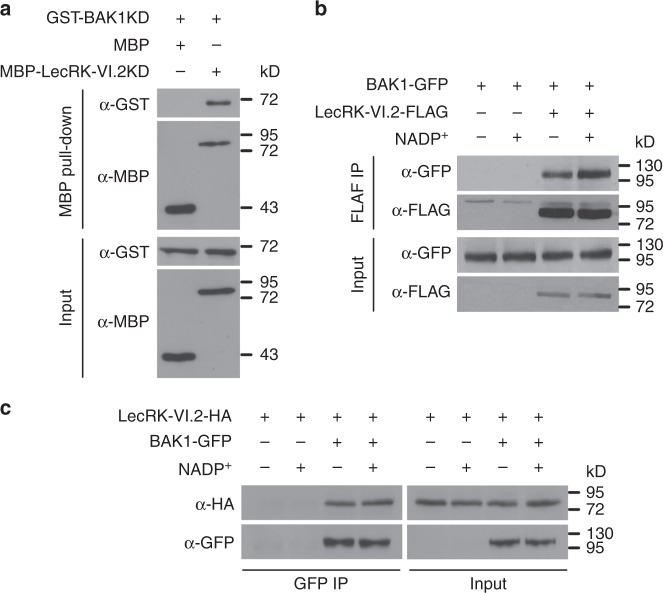
Physical association between LecRK-VI.2 and BAK1. **a** In vitro maltose-binding protein (MBP) pull-down assay of LecRK-VI.2KD interaction with BAK1KD. Recombinant MBP or MBP-LecRK-VI.2KD was incubated with GST-BAK1KD and pulled down with amylose resin beads. Input and bead-bound proteins were analyzed by immunoblotting with monoclonal anti-MBP and anti-GST antibodies. **b** Co-immunoprecipitation (Co-IP) analysis of LecRK-VI.2-FLAG association with BAK1-GFP in *N. benthamiana*. Total proteins (input) of *N. benthamiana* leaves transiently co-expressing LecRK-VI.2-FLAG and BAK1-GFP, or transiently expressing BAK1-GFP alone, were immunoprecipitated with anti-FLAG affinity agarose beads and the precipitates were analyzed by immunoblotting with monoclonal anti-FLAG and anti-GFP antibodies. The leaves were treated with (+) or without (−) 0.8 mm NADP^+^ for 10 min. **c** Co-IP analysis of LecRK-VI.2-HA association with BAK1-GFP in *Arabidopsis*. Total proteins of *Arabidopsis* leaves from the *BAK1:BAK1-GFP*/*LecRK-VI.2:LecRK-VI.2-HA* transgenic plants were immunoprecipitated with monoclonal anti-GFP antibody/protein G plus agarose and the precipitates were analyzed by immunoblotting with polyclonal anti-GFP and monoclonal anti-HA antibodies. The leaves were treated with (+) or without (−) 0.8 mm NADP^+^ for 10 min

As both LecRK-VI.2 and BAK1 function in eNAD(P)^+^ signaling and biological induction of SAR, they may form a protein complex. To test this hypothesis, we examined the interaction between the two proteins using three different approaches. First, we used pull-down assays to investigate possible interaction between the cytoplasmic kinase domains (KDs) of LecRK-VI.2 and BAK1 (LecRK-VI.2KD and BAK1KD, respectively). As shown in Fig. 6a and Supplementary Fig. 8a, amylose beads pulled down both MBP-LecRK-VI.2KD and GST-BAK1KD from a mixture of purified MBP-LecRK-VI.2KD and GST-BAK1KD, but only pulled down MBP from a mixture of purified MBP and GST-BAK1KD or MBP-LecRK-VI.2KD from a mixture of purified MBP-LecRK-VI.2KD and GST. This result indicates that LecRK-VI.2KD can physically interact with BAK1KD in vitro. Second, we employed the co-immunoprecipitation (Co-IP) technique in *N. benthamiana* to test possible association of LecRK-VI.2 with BAK1 in vivo. As shown in Fig. 6b, anti-FLAG beads precipitated both LecRK-VI.2-FLAG and BAK-GFP (green fluorescence protein) from *N. benthamiana* transiently co-expressing the LecRK-VI.2-FLAG and BAK1-GFP fusion proteins, and NADP^+^ treatment did not affect the amount of precipitated BAK1-GFP. In contrast, anti-FLAG beads did not precipitate BAK1-GFP from *N. benthamiana* transiently expressing BAK1-GFP only (Fig. 6b), indicating that BAK1-GFP did not bind nonspecifically to the anti-FLAG beads. Similarly, anti-FLAG beads precipitated LecRK-VI.2-FLAG and BAK1-GFP but not plasma membrane localized LTI6B-GFP^53^ (Supplementary Fig. 8b), excluding the possibility of nonspecific interaction with GFP. These results indicate that LecRK-VI.2-HA and BAK1-GFP constitutively associate with each other when transiently expressed in *N. benthamiana*. Finally, we created stable transgenic *Arabidopsis* plants co-expressing LecRK-VI.2-HA and BAK1-GFP by transforming a *LecRK-VI.2:LecRK-VI.2-HA* construct into the previously characterized *BAK1:BAK1-GFP* plants^54^. The *LecRK-VI.2:LecRK-VI.2-HA* construct was also transformed into wild type to generate plants expressing LecRK-VI.2-HA only as a control. Co-IP experiments were conducted using these plants to test the association between LecRK-VI.2-HA and BAK1-GFP. As shown in Fig. 6c, anti-GFP/protein G plus agarose precipitated both BAK1-GFP and LecRK-VI.2-HA from the *BAK1:BAK1-GFP*/*LecRK-VI.2:LecRK-VI.2-HA* plants, and NADP^+^ treatment did not increase the amount of LecRK-VI.2-HA. On the other hand, anti-GFP/protein G plus agarose did not precipitate any lecRK-VI.2-HA from plants carrying the *LecRK-VI.2:LecRK-VI.2-HA* transgene only. Taken together, these results demonstrate that LecRK-VI.2 and BAK1 constitutively form a protein complex in vivo.

To confirm that LecRK-VI.2 and BAK1 function through a protein complex in eNAD(P)^+^ signaling and SAR, we generated a *lecrk-VI.2 bak1* double mutant by crossing *lecrk-VI.2-2* with *bak1-5* and tested its responses to exogenous NADP^+^ treatment and biological induction of SAR. As shown in Supplementary Fig. 9a, b, NADP^+^-induced and SAR activation-conferred resistance to *Psm* was comparable in the *lecrk-VI.2 bak1* double mutant and the *lecrk-VI.2-2* single mutant. This result indicates no additive effect between the *lecrk-VI.2-2* and *bak1-5* mutations, supporting that LecRK-VI.2 and BAK1 function in SAR by constituting a protein complex.

### The importance of the kinase activity of LecR-VI.2 and BAK1

**Fig. 7:**
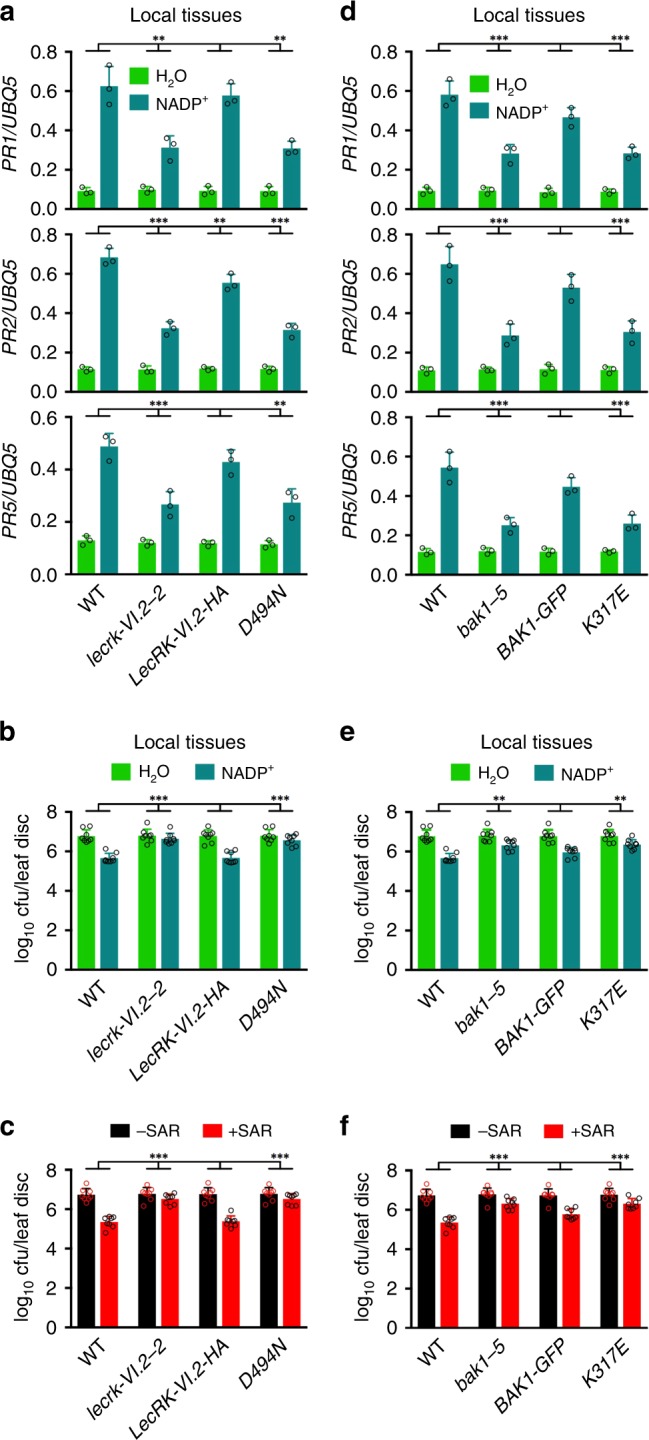
The necessity of the kinase activities of LecRK-VI.2 and BAK1 in eNADP^+^ signaling and SAR. **a** Exogenous NADP^+^-induced local expression of *PR1*, *PR2*, and *PR5* in the wild-type (WT), *lecrk-VI.2-2*, *LecRK-VI.2:LecRK-VI.2-HA*, and *LecRK-VI.2:lecrk-VI.2(D494N)-HA* plants. Leaves of 4-week-old soil-grown plants were infiltrated with 0.4 mm NADP^+^ or H_2_O. The infiltrated leaves were collected 20 hr later. Total RNA was extracted and subjected to qPCR analysis. Expression levels were normalized against the constitutively expressed *UBQ5*. Data represent the mean ± SD of three biological replicates. Asterisks denote significant differences between the induction in the mutant or transgenic plants and that in the wild type (**p < 0.01, ***p < 0.001; two-way ANOVA with Sidak’s test). **b** Exogenous NADP^+^-induced local resistance in the wild-type, *lecrk-VI.2-2*, *LecRK-VI.2:LecRK-VI.2-HA*, and *LecRK-VI.2:lecrk-VI.2(D494N)-HA* plants. Leaves of 4-week-old soil-grown plants were infiltrated with 0.4 mm NADP^+^ or H_2_O. Five hr later, the infiltrated leaves were inoculated with a *Psm* suspension (OD_600_ = 0.001). Three d later, eight leaves were collected to examine the growth of the pathogen. Data represent the mean ± SD of eight biological replicates. Asterisks denote significant differences between the induction in the mutant or transgenic plants and that in the wild type (***p < 0.001; two-way ANOVA with Sidak’s test). **c** Biological induction of SAR in the wild-type, *lecrk-VI.2-2*, *LecRK-VI.2:LecRK-VI.2-HA*, and *LecRK-VI.2:lecrk-VI.2(D494N)-HA* plants. Three lower leaves on each 4-week-old soil-grown plant were infiltrated with 10 mm MgCl_2_ or a *Psm* suspension (OD_600_ = 0.002). Two d later, two systemic leaves were challenge-inoculated with *Psm* (OD_600_ = 0.001). Three d later, eight leaves were collected to examine the growth of the pathogen. Data represent the mean ± SD of eight biological replicates. Asterisks denote significant differences between the induction in the mutant or transgenic plants and that in the wild type (***p < 0.001; two-way ANOVA with Sidak’s test). **d** Exogenous NADP^+^-induced local expression of *PR1*, *PR2*, and *PR5* in the wild-type, *bak1-5*, *35* *S:BAK1-GFP*, and *35* *S:bak1(K317E)-GFP* plants. The experiment was conducted as in **a**. Data represent the mean ± SD of three biological replicates. Asterisks denote significant differences between the induction in the mutant or transgenic plants and that in the wild type (***p < 0.001; two-way ANOVA with Sidak’s test). **e** Exogenous NADP^+^-induced local resistance in the wild-type, *bak1-5*, *35* *S:BAK1-GFP*, and *35* *S:bak1(K317E)-GFP* plants. The experiment was conducted as in **b**. Data represent the mean ± SD of eight biological replicates. Asterisks denote significant differences between the induction in the mutant or transgenic plants and that in the wild type (**p < 0.01; two-way ANOVA with Sidak’s test). **f** Biological induction of SAR in the wild-type, *bak1-5*, *35* *S:BAK1-GFP*, and *35* *S:bak1(K317E)-GFP* plants. The experiment was conducted as in **c**. Data represent the mean ± SD of eight biological replicates. Asterisks denote significant differences between the induction in the mutant or transgenic plants and that in the wild type (***p < 0.001; two-way ANOVA with Sidak’s test)

To determine whether phosphorylation is involved in LecRK-VI.2/BAK1-mediated signaling, we tested if the kinase activities of LecRK-VI.2 and BAK1 are required for their functions in eNADP^+^ signaling and biological induction of SAR. To this end, a point mutation was introduced into the *LecRK-VI.2:LecRK-VI.2-HA* construct to replace a conserved Asp (D) residue at the position 494 of the catalytic loop HRD motif with an Asn (N) residue. The resulting construct (*LecRK-VI.2:lecrk-VI.2(D494N)-HA*) and the wild-type construct were then transformed into the *lecrk-VI.2-2* background. As shown in Fig. 7a–c and Supplementary Fig. 10a, the wild-type construct *LecRK-VI.2:LecRK-VI.2-HA* almost completely complemented the defects of *lecrk-VI.2-2* in NADP^+^-induced *PR* gene expression and *Psm* resistance as well as biological induction of SAR, whereas the *LecRK-VI.2:lecrk-VI.2(D494N)-HA* construct did not rescue any of these defects. For BAK1, we transformed the *35* *S:BAK1-GFP* construct and a *35* *S:bak1(K317E)-GFP* construct into the *bak1-5* background. The *K317E* mutation has been shown to abolish the kinase activity of BAK1^54,55^. Although it has been shown that BAK1-GFP is not fully functional in PTI responses and *bak1-5* is a dominant-negative mutation^49,56^, the *35* *S:BAK1-GFP* construct largely complemented the *bak1-5* mutant phenotypes including reduced induction of *PR* gene expression and *Psm* resistance by NADP^+^ as well as compromised SAR (Fig. 7d–f and Supplementary Fig. 10b). To exclude the possibility of seed contamination or transgene modification, we confirmed the *bak1-5* genetic background of the transgenic plants and the inability of BAK1-GFP to complement *bak1-5* in flg22-induced ROS burst (Supplementary Fig. 11a, b). Therefore, C-terminal-tagged BAK1 is still functional in NADP^+^ signaling and SAR. On the other hand, the *35* *S:bak1(K317E)-GFP* construct did not complement any of the *bak1-5* phenotypes (Fig. 7d–f and Supplementary Fig. 10b). Taken together, these results demonstrate that the kinase activities of both LecRK-VI.2 and BAK1 are required for eNADP^+^-induced immune responses and biological induction of SAR, suggesting that phosphorylation is involved in LecRK-VI.2/BAK1-mediated eNADP^+^ and SAR signaling.

### LecRK-VI.2 is required for flg22-induced systemic immunity

It has been shown that LecRK-VI.2 contributes to PTI responses including flg22 (a 22-amino acid peptide corresponding to the N terminus of bacterial flagellin)-induced MPK3/6 activation, callose deposition, and stomatal closure^25^. Consistently, the *lecrk-VI.2-1* mutant plants are highly sensitive to *P. syringae* pv. *tomato* DC3000 when plants are dip inoculated^25^. To explore possible relationship between eNAD(P)^+^ and flg22, we tested flg22-induced local apoplastic and systemic resistance in the eNAD(P)^+^ receptor mutants. To this end, we first generated a *lecrk-I.8 lecrk-VI.2* (*lecrkI.8/VI.2*) double mutant by crossing *lecrk-I.8-2* with *lecrk-VI.2-2* and tested its responses to exogenous NAD(P)^+^ treatment and biological induction of SAR. The double mutant behaved like *lecrk-I.8-2* upon NAD^+^ treatment and like *lecrk-VI.2-2* upon NADP^+^ treatment or SAR induction (Supplementary Fig. 12a-c), suggesting that mutations in *LecRK-I.8* and *LecRK-VI.2* inhibit eNAD^+^ and eNADP^+^ signaling, respectively. We then tested flg22-induced local apoplastic resistance in the double mutant. As shown in Supplementary Fig. 12d, 100 nm flg22 induced similar levels of resistance to *Psm* in the wild-type, *lecrk-I.8-2*, *lecrk-VI.2-2*, and *lecrkI.8/VI.2* plants. Finally, we examined flg22-induced systemic immunity in the *lecrk-VI.2* single mutants, as the *lecrk-I.8-2* mutation did not affect SAR^22^ (Supplementary Fig. 12c). To this end, three lower leaves on each plant were infiltrated with 200 nm flg22 every 12 hours for a total of 4 times. Five hours after the last infiltration, the systemic leaves were challenge-inoculated with *Psm* to test flg22-induced resistance. As shown in Supplementary Fig. 12e, flg22-induced systemic resistance was significantly inhibited in the three *lecrk-VI.2* mutants. Therefore, LecRK-VI.2 is crucial for flg22-induced systemic immunity but not for flg22-induced local apoplastic resistance.

## Discussion

Although it has been well documented in animals that eNAD(P)^+^ functions in numerous physiopathological processes^18^, eNAD(P)^+^ and its signaling role in plants have not been established. The identification of the first eNAD^+^-binding receptor in *Arabidopsis* has stimulated us to begin investigating this interesting molecule in depth^22^. Here, we identified LecRK-VI.2 as a potential primary eNAD(P)^+^-binding receptor, which allowed us to uncover the LecRK-VI.2/BAK1 complex that has a central role in the establishment of SAR in *Arabidopsis*.

Several lines of evidence generated in this study suggest that eNAD(P)^+^ may be an SAR signal molecule in *Arabidopsis*. First, NAD(P)^+^ continuously leaks into the extracellular space during pathogen infection (Fig. 1a, b). Second, local application of physiological concentrations of NAD(P)^+^ induces resistance in the systemic leaves (Fig. 1c, d). Third, mutations in the potential eNAD(P)^+^-binding receptor LecRK-VI.2 significantly inhibit SAR induction (Fig. 4). And fourth, BAK1 and BKK1 are required for both eNAD(P)^+^ signaling and SAR (Fig. 5). The above hypothesis is also supported by our previous finding that removal of eNAD(P)^+^ by transgenic expression of the human NAD(P)^+^-hydrolyzing ectoenzyme CD38 partially compromises biological induction of SAR^21^.

Interestingly, locally applied ^32^P-NAD^+^ and/or its metabolite(s) moved systemically, although the radioactivity signal detected in the systemic leaves is much weaker than that in the local leaves (Fig. 1e, f). Approximately 10.8% of the applied radioactivity moved into the systemic leaves in wild-type *Arabidopsis* plants (Supplementary Fig. 2a), suggesting that only a small fraction of eNAD(P)^+^ accumulated in the local leaves could be transported to the systemic leaves. Similarly, only a small fraction (~7–10%) of several other putative SAR mobile signals including AzA and G3P is transported from the local to systemic leaves^57^. Thus, eNAD(P)^+^ could potentially function as an SAR mobile signal in plants. However, as exogenously added molecules often move systemically^58,59^, further in-depth investigations are required to conclusively determine whether and how endogenous eNAD(P) ^+^ moves.

Treatment of various SAR mutants revealed that eNAD(P)^+^ functions either downstream or independently of the putative mobile signals Pip, NHP, G3P, and AzA in both local and systemic leaves (Fig. 3c and Supplementary Fig. 4a). It has been proposed that Pip, NO, ROS, AzA, and G3P function in a signaling amplification loop and that ROS mediates a systemic signal network in SAR^7,15,16^. Biochemically, ROS has been shown to oxidize cell membrane, leading to pore formation and membrane disintegration^60,61^. Thus, the ROS produced in the Pip-NO-ROS-AzA-G3P amplification loop may cause reversible or irreversible injuries to the plasma membrane, resulting in leakage of cellular NAD(P)^+^ into the extracellular space to activate its receptor LecRK-VI.2. On the other hand, eNAD(P)^+^-induced defense signaling requires SA (Fig. 3c and Supplementary Fig. 4a), and, conversely, SA induces *LecRK-VI.2* gene expression through the coactivator NPR1 (Supplementary Fig. 5c). These results suggest a signaling amplification loop involving SA, NPR1, and LecRK-VI.2. Nevertheless, as NAD(P)^+^-induced expression of *PR1*, an SA pathway marker gene, is not always correlated with NAD(P)^+^-induced disease resistance (Fig. 3a, b and 5a, b), the relationship between eNAD(P)^+^ and SA as well as other SAR signal molecules is complicated and warrants further investigation.

LecRK-VI.2 binds both NAD^+^ and NADP^+^ (Fig. 2e–h), indicating that LecRK-VI.2 is a potential receptor for both NAD^+^ and NADP^+^. Interestingly, mutations in *LecRK-VI.2* did not affect NAD^+^-induced resistance to *Psm* in the local leaves^22^, but completely blocked both NAD^+^- and NADP^+^-induced resistance in the systemic leaves (Fig. 3b), suggesting that LecRK-VI.2 may be involved in eNAD(P)^+^-triggered SAR signal production in the local leaves and/or perceiving eNAD(P)^+^ and/or other signal molecules in the systemic leaves. Furthermore, while exogenous NAD(P)^+^-induced systemic *PR1* transcription was only slightly reduced in *lecrk-VI.2* mutants (Fig. 3a), *Psm*-induced systemic *PR* gene expression was almost completely abolished (Fig. 4a–c), suggesting that LecRK-VI.2 may have eNAD(P)^+^-independent functions. This is not without precedent for PRRs. For instance, the human cell-surface receptors TLR2 (Toll-like receptor 2) and TLR4 recognize a group of chemically different ligands including PAMPs and DAMPs^62^. Further investigation on potential ligands of LecRK-VI.2 would help reveal the activation mechanisms of this important plant immune regulator.

Mutations in *LecRK-VI.2* had no effect on basal immunity against bacterial pathogens when the bacteria were inoculated by leaf infiltration^25^, but significantly suppressed biological induction of SAR (Fig. 4). The partial SAR defect in *lecrk-VI.2* mutants may be attributed to possible redundancy in eNAD(P)^+^ perception, as the *Arabidopsis* genome encodes 75 LecRKs (32 G-type, 42 L-type, and 1 C-type)^63^. Indeed, LecRK-VI.2 is not the only receptor for eNAD(P)^+^ in *Arabidopsis*^22^. Nevertheless, ~ 90% of the transcriptome changes in the systemic leaves were suppressed in the *lecrk-VI.2-2* mutant (Fig. 4e, f), indicating that LecRK-VI.2 is a key SAR signaling component responsible for the vast majority of SAR signaling triggered by *Psm*. In line with this conclusion, overexpression of *LecRK-VI.2* has been shown to confer constitutive resistance against bacterial pathogens, which is a phenotype similar to SAR^25^. As LecRK-VI.2 is a plasma membrane-located transmembrane protein with an extracellular NAD(P)^+^-binding domain and a cytoplasmic kinase domain (Fig. 2e–h)^64,65^, these results suggest that eNAD(P)^+^-triggered LecRK-VI.2-mediated outside-in signaling is an integral constituent of the SAR signaling pathway. In support of this hypothesis, the LecRK-VI.2-associating BAK1 and its homolog BKK1, two plasma membrane-located LRR-RKs^50,54,55^, are also required for SAR (Fig. 5c, d).

BAK1 and BKK1 have been established as crucial co-receptors or adaptors for a number of LRR domain PRRs^66^. The PRR-BAK1 immune complex formation is fine-tuned by other regulators. For instance, the malectin-like RLKs FERONIA (FER) and IMPAIRED OOMYCETE SUSCEPTIBILITY1 constitutively associate with both FLS2 and BAK1 to act as scaffolds for ligand-induced FLS2-BAK1 complex formation^67,68^. FER is a receptor for several *Arabidopsis* RAPID ALKALINIZATION FACTORs (RALFs)^68,69^. The constitutive association between BAK1 and FER can be strongly enhanced upon treatment with flg22, whereas binding of RALF23 to FER inhibits flg22-induced complex formation between FLS2 and BAK1^68^. LecRK-VI.2 also constitutively associates with FLS2 and BAK1^70^ (Fig. 6), but is dispensable for flg22-induced FLS2-BAK1 complex formation, suggesting a distinct mechanism underlying the function of LecRK-VI.2 in PTI. As a potential eNAD(P)^+^ receptor, binding of eNAD(P)^+^ may enhance its function in the FLS2-BAK1 immune complex. Indeed, exogenous NAD^+^ induces expression of the PTI marker genes *WRKY53* and *FRK1* as well as production of ROS^20,22^.

The interplay between eNAD(P)^+^ and flg22 (and other PAMPs) signaling is complex and deserves further attention. Recognition of flg22 by FLS2 results in PTI responses including ROS burst, which could potentially damage the plasma membrane, contributing to accumulation of eNAD(P)^+^. eNAD(P)^+^ in turn could potentiate flg22 signaling through the LecRK-VI.2-FLS2 complex. Indeed, flg22-induced MPK3/6 activation, callose deposition, and stomatal closure are inhibited in *lecrk-VI.2-*mutant plants^25^, though the contribution of eNAD(P)^+^ in this process remains to be determined. On the other hand, the potential roles of eNAD(P)^+^ in PAMP signaling and SAR are clearly distinct. First, only BAK1 and BKK1, but not FLS2, are required for SAR (Fig. 5c, d), indicating that the LecRK-VI.2-BAK1 complex, but not the LecRK-VI.2-FLS2 complex, has a major role in SAR. In agreement with this idea, flg22-induced systemic immunity is compromised in the *lecrk-VI.2* mutants, whereas flg22-induced local apoplastic resistance is not significantly affected by the mutations (Supplementary Fig. 12d, e). Furthermore, the BAK1-GFP fusion is not functional in PTI responses^56^, but is active in NAD(P)^+^ signaling and SAR (Fig. 7d–f), indicating that the LecRK-VI.2-BAK1 complex-mediated eNAD(P)^+^ signaling is likely independent or downstream of flg22 signaling.

The kinase activities of both LecRK-VI.2 and BAK1 are essential to their function in eNADP^+^ signaling and SAR (Fig. 7), suggesting that binding of eNAD(P)^+^ to LecRK-VI.2 may trigger transphosphorylation between LecRK-VI.2 and BAK1, which in turn may phosphorylate downstream target(s). A potential target is the serine/threonine kinase SNF1-RELATED PROTEIN KINASE2.8 (SnRK2.8), which has been shown to function in SAR via phosphorylating NPR1^71^. NPR1 is a master transcription coactivator in plants and nuclear localization is required for its function in plant immunity^33^. In the systemic leaves, SAR activation-induced redox changes switch NPR1 from an oligomer to monomers and SnRK2.8 phosphorylates monomeric NPR1 at Ser-589 and Thr-373 to facilitate its nuclear import, which is a critical step in SAR induction^71^.

Induction of SAR comprises four stages: signal generation, signal translocation, signal perception, and SAR execution. Among the four stages, signal perception in the systemic leaves is the least understood^3,24^. In this study, we uncovered a putative SAR mobile signal eNAD(P)^+^ and its potential receptor LecRK-VI.2 and demonstrate that the LecRK-VI.2/BAK1 complex has a pivotal role in SAR. Identification of the precise relationship between eNAD(P)^+^ and other SAR signal molecules as well as the downstream targets of the LecRK-VI.2/BAK1 complex will shed further light on the SAR signaling process.

## Methods

### *Arabidopsis* growth

*Arabidopsis thaliana* seeds were sown on autoclaved soil (Sunshine MVP; Sun Gro Horticulture, Agawam, MA, USA) and cold-treated at 4 °C for 3 days. After germination, seedlings were grown at 22–24 °C under a 16-hr-light/8-hr-dark regime for 2 weeks. Seedlings were then transplanted individually into pots containing the autoclaved soil. Four-week-old plants were used for NAD(P)^+^ treatment and *Psm* inoculation.

### *Pseudomonas* culture

*Pseudomonas syringae* pv. *maculicola* ES4326 (*Psm*) was grown at 28 °C in King’s B medium containing 50 μg mL^−1^ streptomycin under shaking at 220 rpm. Bacteria in overnight log-phase cultures were precipitated by centrifugation at 500 × *g* for 5 min, re-suspended in 10 mm MgCl_2_, and diluted to different final OD_600_ levels for leaf infiltration experiments.

### NAD(P) leakage

Three fully expanded leaves on each 4-week-old soil-grown *Arabidopsis* plant were infiltrated from the abaxial side with 10 mm MgCl_2_ (mock) or a *Psm* suspension (OD_600_ = 0.002). Ten min after the treatment, 30 leaf disks (~7 mm in diameter) for each treatment were removed and washed twice for a total of 50 min. Sets of 10 leaf disks each were then placed in 5 mL fresh water in a test tube. NAD(P) concentrations in the water were measured over time following an enzymatic cycling assay protocol. One hundred μL of the water surrounding the leaf disks was added into a 1.5 mL microcentrifuge tube containing 0.1 mL Bicine-NaOH buffer (1.0 m, pH 8.0), followed by addition of 0.1 mL each of EDTA (disodium salt, 40 mm), 3-(4,5-dimethylthiazolyl-2)-2,5-diphenyltetrazolium bromide (MTT, 4.2 mm), phenazine ethosulfate (PES, 16.6 mm), and ethanol (5.0 m) for the determination of NAD or 0.05 mL of glucose-6-phosphate (G6P, 50 mm) for the determination of NADP. After adjusting the total volume to 1 mL by adding H_2_O, the microcentrifuge tube was kept at 37 °C for 5 min. The reaction was started by adding 0.02 mL of alcohol dehydrogenase (500 units mL^−1^, for NAD) or G6P dehydrogenase (35 units mL^−1^, for NADP). After incubated at 37 °C for 10 min, the reaction mixture was transferred to a microcuvette and the absorbance at 570 nm was measured with a Beckman Du640 spectrophotometer (Beckman).

### Plant treatment with NAD^+^ and NADP^+^

β-Nicotinamide adenine dinucleotide sodium salt (NAD^+^-Na; Sigma-Aldrich) and β-NAD^+^ phosphate sodium (NADP^+^-Na; Sigma-Aldrich) were dissolved in water, and the pH of the resulting solutions was adjusted to ~6.0 using 0.1 m NaOH. The solution (0.2 mm NAD^+^ or 0.4 mm NADP^+^) was infiltrated from the abaxial side into *Arabidopsis* leaves of 4-week-old soil-grown plants using a 1-mL needleless syringe. Distilled water was used as the negative control. The infiltration was conducted between 10 and 11 AM. For NAD(P)^+^-induced defense gene expression, the infiltrated leaf tissues were collected 20 hr after the treatment for RNA analysis. For NAD(P)^+^-induced *Psm* resistance, the infiltrated leaves were inoculated with *Psm* 5 hr after the NAD(P)^+^ treatment as described below (“assessment of plant resistance to *Psm*”). For SAR induction, three lower leaves on each *Arabidopsis* plant were infiltrated with H_2_O (mock), 0.4 mm NAD^+^, or 0.8 mm NADP^+^ every 12 hr for a total of four times. The first infiltration was performed at around 9 PM. About 5 hr after the last infiltration, two systemic (upper untreated) leaves were either collected for defense gene analysis or challenge-inoculated with *Psm* as described below (“assessment of plant resistance to *Psm*”).

### Biological induction of SAR

Three lower leaves on each 4-week-old soil-grown *Arabidopsis* plant were infiltrated from the abaxial side with 10 mm MgCl_2_ (mock) or a *Psm* suspension (OD_600_ = 0.002). The SAR activation treatment was conducted between 10 and 11 AM. Two days later, two systemic leaves were either collected for defense gene analysis or challenge-inoculated with *Psm* as described below (“assessment of plant resistance to *Psm*”).

### Assessment of plant resistance to *Psm*

To assess *Psm* resistance in the NAD(P)^+^-treated leaves or the systemic leaves, a *Psm* suspension (OD_600_ = 0.001) was infiltrated from the abaxial side into the target leaves using a 1-mL needleless syringe. A total of 12 plants were used per genotype/treatment. Two and a half to three days later, eight leaves per genotype/treatment from eight plants (one leaf from each plant) were collected to examine the growth of the pathogen. A leaf disk (~7 mm in diameter) was removed from each leaf using a hole punch and the leaf disk was placed into 500 μL of 10 mM MgCl_2_ in a 2-mL microcentrifuge tube and ground forcefully using a plastic pestle. Twenty-fold serial dilutions of the homogenate were plated on trypticase soy agar (BD) plates supplemented with 50 μg/mL streptomycin to determine bacterial concentrations. Bacterial growth rates were expressed as colony-forming units (cfu) per leaf disk area. All experiments depicted in the figures were repeated at least three times with similar results.

### RNA analysis

For reverse transcription (RT), total RNA was treated with DNase I (ThermoFisher Scientific) at 37 °C for 30 min. After inactivation of the DNase, RT was performed using SUPERSCRIPT First-strand Synthesis System (ThermoFisher Scientific) and 2 μg of the DNase-treated RNA in a 20 μL reaction. Quantitative PCR was performed using SYBR Green protocol (Applied Biosystems) with 1 μm primers and 0.2 μL aliquot of RT product in a total of 12.5 μL per reaction. Reactions were run and analyzed on a MX3000P real-time PCR machine (Stratagene) according to the manufacturer’s instructions. A standard curve was made by determining the threshold cycle (Ct) values for a dilution series of the RT reaction product for each primer pair. For each reaction, the Ct was determined by setting the threshold within the logarithmic amplification phase. The relative quantity of a gene is expressed in relation to *UBIQUITIN5* (*UBQ5*) using the formula 2^(Ct^*UBQ5*^-Ct^*GENE*^), where 2 represents perfect PCR efficiency. All experiments described in the figures were repeated at least three times with similar results.

### NAD^+^ movement assay

Three lower leaves on a 4-week-old soil-grown *Arabidopsis* plant or two lower leaves on a 5-week-old soil-grown *N. benthamiana* plant were infiltrated from the abaxial side with a water solution of 1 mm unlabeled NAD^+^ plus 6.25 nm ^32^P-NAD^+^ (specific activity 800 Ci/mmol; PerkinElmer, USA). The treated plants were incubated at 22–24 °C under a 16-hr-light/8-hr-dark regime for 24 hr. The infiltrated leaves and two systemic leaves from each plant were collected, wrapped with plastic waterproofing membrane, and exposed to X-ray film for 24 hr at −80 °C. The X-ray film was then developed to visualize the radioactivity in the leaves.

### Protein purification

For purification of MBP, MBP-eLecRK-VI.2, and MBP-eDORN1, a single colony of BL21(DE3) carrying the corresponding plasmid was cultured overnight at 37 °C in 5 mL Lysogeny broth (LB) with 50 µg/mL ampicillin. One mL of the seed culture was added to 500 mL fresh LB medium with 50 µg/mL ampicillin and cultured at 37 °C with shaking to an OD_600_ of 0.4. Isopropyl *β*-d-1-thiogalactopyranoside was added to a final concentration of 0.3 mm and the culture was allowed to grow for another 16–18 hr at 18 °C before the cells were harvested for protein extraction. MBP-fusion proteins were purified with amylose resin according to the protocol supplied by the manufacturer (New England BioLabs).

### NAD^+-^binding assays

Amylose resin beads with the bound proteins were re-suspended in binding buffer (10 mm HEPES, pH 7.5, 5 mm MgCl_2_) and aliquoted in 86 µL portions for individual binding reactions. For total binding assay, 10 µL binding buffer and 4 µL ^32^P-NAD^+^ (6.25 µm) were added, resulting in 250 nm ^32^P-NAD^+^ in the final 100 µL reaction mixture. For nonspecific binding, 10 µL of 2.5 mm unlabeled NAD^+^ and 4 µL ^32^P-labeled NAD^+^ were added, resulting in 250 µm unlabeled NAD^+^ and 250 nm ^32^P-NAD^+^ in the final 100 µL reaction mixture. The mixtures were incubated for 30 min at room temperature with gentle mixing every 5 min. The beads containing the binding reactions were then precipitated by centrifugation at 500 × *g* for 5 min, washed three times with the binding buffer, re-suspended in 10 mL ScintiVerse BD Cocktail (ThermoFisher Scientific) per sample, and carefully transferred into scintillation vials. The vials were placed in a Beckman Coulter LS6500 Multi-Purpose Scintillation Counter (Beckman) and bound ^32^P-NAD^+^ was quantified by scintillation counting. Specific NAD^+^ binding was calculated by subtracting the nonspecific binding from the total binding. For saturation binding assay, amylose resin beads with the bound proteins were incubated with different concentrations (50, 200, 500, 1000, 1500, and 2000 nm) of ^32^P-NAD^+^ in the absence (for total binding) or presence (for nonspecific binding) of additional 1000-fold unlabeled NAD^+^ in the binding buffer. The dissociation constant (*K*d) was calculated by one site specific binding saturation model using GraphPad Prism 7 (GraphPad Software, La Jolla, CA, USA). For competitive binding assay, amylose resin beads with the bound proteins were incubated with 250 nm of ^32^P-labeled NAD^+^ in the presence of different concentrations (100 nm, 1 µm, 10 µm, 100 µm, and 1 mm) of unlabeled nucleotides (NAD^+^, NADP^+^, and ATP) in the binding buffer. The 50% inhibition concentration (IC_50_) values were calculated by importing the data points into GraphPad Prism 7 using the one site Fit logIC_50_ competition model.

For microsome-based binding assays, *Arabidopsis* plants were grown under a 15-hr-light/9-hr-dark regime for about 6 weeks. Rosette leaf tissues were homogenized with a mortar and pestle in 1 mL/1 gram chilled membrane extraction buffer (20 mm Tris-HCl, pH 7.5, 250 mm mannitol, 5 mm MgCl_2_, 0.1 mm CaCl_2_, and protease inhibitors). Homogenates were filtered through two layers of Miracloth and centrifuged at 10,000 × *g* for 10 min at 4 °C. The supernatant was centrifuged at 100,000 × *g* for 1 hr at 4 °C to pellet microsomes. The microsomal pellet was re-suspended at a protein concentration of 2 mg/mL in binding buffer (10 mm MES-KOH, pH 5.7, 5 mm MgCl_2_, 0.25 mm CaCl_2_, 0.25 m mannitol, and protease inhibitors) and aliquoted in 10 µL portions for individual binding reactions. For total binding assay, 4 µL binding buffer and 1 µL ^32^P-NAD^+^ were added, resulting in ~420 nm ^32^P-NAD^+^ in the final 15 µL reaction mixture. For competitive binding assay, the microsomes were incubated with 420 nm ^32^P-NAD^+^ in the presence of 420 µm unlabeled NAD^+^ or NADP^+^ in the binding buffer. The binding reactions were incubated for 30 min at room temperature with gentle mixing every 5 min. The bound and free ^32^P-NAD^+^ were separated by filtering the mixture through a glass-fiber filter (GE Healthcare) and washing with 10 mL ice-cold binding buffer, and were quantified by scintillation counting.

### Microarray analysis

Three lower leaves on each four-week-old soil-grown plant were infiltrated with either 10 mm MgCl_2_ (−SAR) or *Psm* (OD_600_ = 0.002) (+SAR). Two days later, the systemic leaves were collected for total RNA extraction. Total RNA samples were subjected to microarray analysis using the Affymetrix microarray platform, which was performed at Interdisciplinary Center for Biotechnology Research Gene Expression and Genotyping Core at The University of Florida. RNA concentration was determined on a NanoDrop Spectrophotometer (ThermoFisher Scientific) and sample quality was assessed using the Agilent 2100 Bioanalyzer (Agilent Technologies). All microarray sample preparation used the GeneChip 3’ IVT Plus Express kit (ThermoFisher Scientific), and reactions were done following the manufacturer’s protocols. In brief, cDNA was synthesized from 500 ng of total RNA and used as template for in vitro transcription during which a biotin-modified nucleotide was incorporated. The biotin-labeled aRNA (amplified RNA) was then purified and fragmented. Eleven μg of biotin-labeled aRNAs were hybridized with rotation at 45 °C for 16 hr to the GeneChip Arabidopsis Genome ATH1 Array (ThermoFisher Scientific). The arrays were washed and stained with the reagents supplied in GeneChip Hybridization Wash and Stain kit (ThermoFisher Scientific) on an Affymetrix Fluidics Station 450, and scanned with a GeneChip 7 G Scanner (ThermoFisher Scientific).

The microarray data were pre-processed and normalized using the affy package^72^. The Robust Multichip Analysis approach was applied for the normalization. After normalization, the empirical Bayes moderated *t* statistics, which is implemented in the limma Bioconductor package^73^, was performed for differential expression detection. In each comparison, a *p* value and fold change were computed for each gene locus. The gene expression fold changes were computed based on the normalized log-transformed signal intensity data. To control false discovery rate and correct multiple hypothesis testing, a *q* value was calculated and used to assess the significance of each test using Benjamini and Hochberg’s approach^74^. Genes with an absolute fold change ≥ 2 and a *q* value ≤ 0.05 were considered as significantly induced or suppressed and were then further explored to obtain overlapped genes among genotypes. The venn function implement in limma package was applied to compute classification counts and draw a venn diagram.

### Plasmid construction and plant transformation

For expression of the MBP-eLecRK-VI.2 fusion protein in *E. coli*, the DNA fragment encoding the extracellular domain (AAs 23-310) of LecRK-VI.2 was amplified using the primers *Xba*I-eLecRK-VI.2 F and *Hin*dIII-eLecRK-VI.2 R (All primers are listed Supplementary Table 1). The PCR products were digested with *Xba*I and *Hin*dIII and cloned into the *Xba*I/*Hin*dIII site of pMAL-p2X, generating pMAL-p2X-eLecRK-VI.2. The pMAL-p2X-eDORN1 plasmid has previously been described^22^. For expression of the MBP-LecRK-VI.2KD (AAs 331–682) and GST-BAK1KD (AAs 250–615) fusion proteins in *E. coli*, DNA fragments encoding the KDs of LecRK-VI.2 and BAK1 were amplified using primers *Bgl*II-LecRK-VI.2KDF/*Xho*I-LecRK-VI.2KDR and EcoRI-BAK1KDF/*Sal*I-BAK1KDR and cloned into the *Bam*HI/*Sal*I site of pMAL-p2X and the *Eco*RI/*Sal*I site of pGEX-4T-1, generating pMAL-p2X-LecRK-VI.2KD and pGEX-4T-BAK1KD, respectively. For transient expression in *N. benthamiana*, the coding regions of *LecRK-VI.2* and *BAK1* were amplified using primers *Xba*I-LecRK-VI.2 F/*Xho*I-lecRK-VI.2-noStopR and SacI-BAK1F/*Sal*I-BAK1-noStopR and cloned into the *Xba*I/*Sal*I site of pCAMBIA1300S-FLAG and the *Sac*I/*Sal*I site of pCAMBIA1300S-GFP, resulting in pCAMBIA1300S-LecRK-VI.2-FLAG and pCAMBIA1300S-BAK1-GFP, respectively. For generation of transgenic *Arabidopsis* plants expressing LecRK-VI.2-HA, the DNA fragment containing the *LecRK-VI.2* promoter and coding region was amplified using primers *Xba*I-LecRK-VI.2PF and *Bgl*II-3×HA-lecRK-VI.2 R and cloned into the *Xba*I/*Bam*HI site of pCB302, resulting in pCB302-LecRK-VI.2:LecRK-VI.2-HA. Site-directed mutagenesis of Asp494 of LecRK-VI.2 and Lys317 of BAK1 was performed in pCB302-LecRK-VI.2:LecRK-VI.2-HA and pCAMBIA1300S-BAK1-GFP, respectively, using primers listed in Supplementary Table 1. The presence of the expected mutations in the resulting constructs was verified by DNA sequencing. The plasmids pCAMBIA1300S-LecRK-VI.2-FLAG, pCAMBIA1300S-BAK1-GFP, pCB302-LecRK-VI.2:LecRK-VI.2-HA, pCB302-LecRK-VI.2:LecRK-VI.2(D494N)-HA, and pCAMBIA1300S-BAK1(K317E)-GFP were introduced into the *Agrobacterium* strain GV3101(pMP90) and pMAL-p2X, pMAL-p2X-eLecRK-VI.2, pMAL-p2X-eDORN1, pMAL-p2X-LecRK-VI.2KD, and pGEX-4T-BAK1KD were introduced into the *E. coli* strain BL21(DE3) by electroporation.

For transient expression in *N. benthamiana*, Agrobacteria carrying pCAMBIA1300S-LecRK-VI.2-FLAG, pCAMBIA1300S-BAK1-GFP were suspended in an induction buffer (10 mm MES-KOH, pH 5.6, 10 mm MgCl_2_ and 100 μm acetosyringone) to an OD_600_ of 0.4, pre-induced for 2 to 3 h at 28 °C, mixed in a 1:1 ratio before infiltration, and then infiltrated from the abaxial side into *N. benthamiana* leaves using a 1-mL needleless syringe. Two to 3 days later, the leaves infiltrated with the Agrobacteria were used for co-immunoprecipitation analysis.

### Pull-down and co-immunoprecipitation assays

One microgram MBP or MBP-LecRK-VI.2KD was incubated with 1 μg GST-BAK1KD in a binding buffer (20 mm Tris-HCl, 200 mm NaCl, 1 mm EDTA, pH 7.4) under agitation at 4 °C. After 2 hr, 50 μL amylose resin beads (New England BioLabs, E8021S) were added, and the incubation continued for another 2 hr. The beads were then washed five times with the washing buffer (20 mm Tris-HCl, 200 mm NaCl, 1 mm EDTA, 0.6% Triton X-100, pH 7.4). Input and pulled down proteins were resolved by 8% sodium dodecyl sulphate-polyacrylamide gel electrophoresis (SDS-PAGE) and detected by immunoblotting using anti-MBP (New England BioLabs, E8032S, dilution 1:10,000) and anti-GST (Cell Signaling, 2625 S, dilution 1:1000) antibodies. Co-immunoprecipitation assay was performed as described by Roux et al.^47^. *N. benthamiana* or *Arabidopsis* leaves were ground in liquid nitrogen and extraction buffer (50 mm Tris-HCl, pH 7.5, 150 mm NaCl, 10% glycerol, 10 mm DTT, 10 mm EDTA, 1 mm NaF, 1 mm Na_2_MoO_4_, 1% [w/v] polyvinylpyrrolidone, 1% [v/v] IGEPAL CA-630, and 1% [v/v] protease inhibitor cocktail) was added at 2 mL/g tissue powder. Samples were clarified by centrifugation at 16,000 × *g* at 4 °C for 20 min. For *N. benthamiana*, the supernatant was incubated with anti-FLAG M2 affinity agarose gel (Sigma-Aldrich, A2220) at 4 °C for 4 hr. For *Arabidopsis*, the supernatant was incubated with monoclonal anti-GFP antibody (Santa Cruz Biotechnology, sc-9996) overnight at 4 °C followed by precipitation with protein G plus agarose (Santa Cruz Biotechnology) for 4 hr. After washing four times with the extraction buffer, proteins were eluted by boiling in 40 μL of 2× Laemmli sample buffer for 10 min. The eluates were separated on 8% SDS-PAGE, transferred onto nitrocellulose membranes (Maine Manufacturing, Gloucester, MA, USA). For *N. benthamiana*, the membranes were probed with monoclonal anti-FLAG (Sigma-Aldrich, F1804, dilution 1:5000) and polyclonal anti-GFP (Santa Cruz Biotechnology, sc-8334, dilution 1:1000) antibodies to detect immunoprecipitated LecRK-VI.2-FLAG and co-immunoprecipitated BAK1-GFP, respectively. For *Arabidopsis*, the membranes were probed with polyclonal anti-GFP and monoclonal anti-HA (Santa Cruz Biotechnology, sc-7392, dilution 1:1000) antibodies to detect immunoprecipitated BAK1-GFP protein and co-immunoprecipitated LecRK-VI.2-HA, respectively.

### Quantification and statistical analysis

Statistical analyses were performed using the data analysis tools (Student’s *t* test) in Microsoft Excel of Microsoft Office 2011 for Macintosh as well as the one-way ANOVA and the two-way ANOVA in Prism 7. Unless indicated otherwise, all experiments were repeated at least three times with similar trends.

### Reporting summary

Further information on research design is available in the Nature Research Reporting Summary linked to this article.

## Supplementary information

Supplementary Information

Description of Additional Supplementary Files

Supplementary Data 1

Reporting Summary

Source Data

## Data Availability

The authors declare that all data supporting the findings of this study are available within the manuscript and its supplementary files or are available from the corresponding author upon request. The source data underlying Figs. 1–7 and Supplementary Figs. 1–12 are provided as a Source Data file. Microarray data generated as part of this study has been deposited in the Gene Expression Omnibus repository under accession code GSE121886.
