# Supplementary material for: Extracellular pyridine nucleotides trigger plant systemic immunity through a lectin receptor kinase/BAK1 complex
Source: Nat Commun. 2019 Oct 22;10:4810. doi: 10.1038/s41467-019-12781-7 (PMC6805918; doi:10.1038/s41467-019-12781-7)
Supplement: Supplementary file 1 — Supplementary Information [file 41467_2019_12781_MOESM1_ESM.pdf]

Supplemental Information

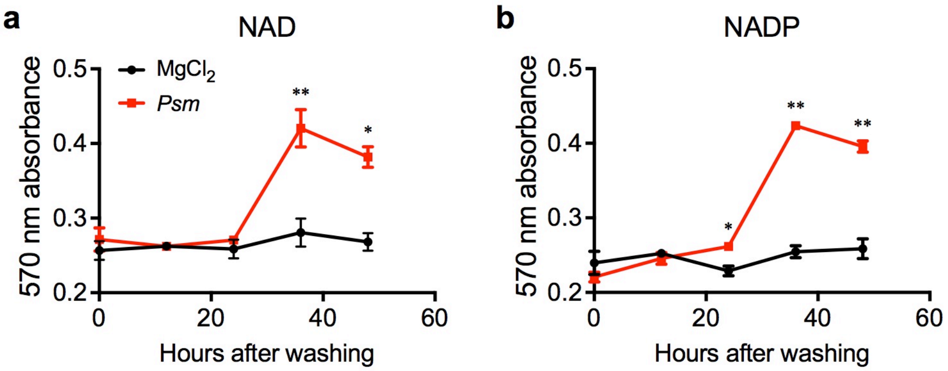

Supplementary Figure 1. NAD and NADP leakage from *Psm*-treated intact leaves

(**a**, **b**) Leaves of 4-week-old soil-grown *Arabidopsis* plants were infiltrated with 10 mM $MgCl_2$ or *Psm* (OD_{600} = 0.002). The infiltrated leaves were removed and sets of 5 leaves were submerged in 2 mL water in test tubes with the petioles above the water surface. NAD (a) and NADP (b) concentrations in the water were measured at the indicated time points by enzymatic cycling assays. Data represent the mean ± standard deviation (SD) of three biological replicates. Asterisks denote significant differences between *Psm*- and $MgCl_2$-treated samples (*$p < 0.05$, **$p < 0.01$; Student's t test).

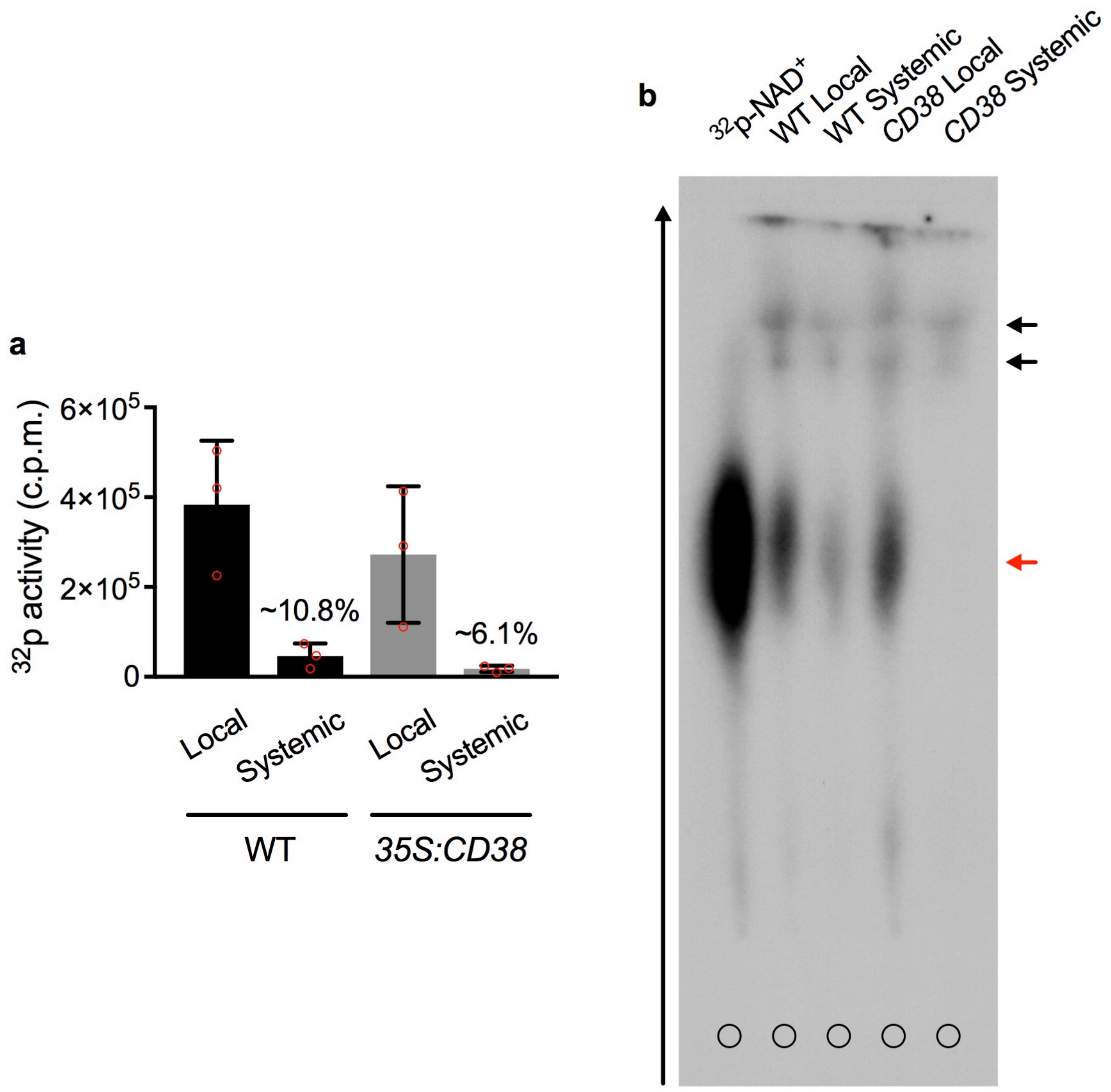

Supplementary Figure 2. Movement of exogenously added ^{32}P-NAD$^+$ in *Arabidopsis* plants

(**a**) Three lower leaves on each *Arabidopsis* wild-type (WT) or *35S:CD38* transgenic plant were infiltrated with a water solution of 6.25 nM ^{32}P-NAD$^+$. Twenty-four hr later, the radioactivity in the local and systemic leaves was quantified. Data represent the mean ± SD of three experiments. The percentages of radioactivity in the systemic leaves were calculated from the following equation: systemic/(local + systemic).

(**b**) Plants were treated as in (a). The local and systemic leaves were submerged in 1 mL 80% ethanol and boiled at 95°C for 2 min. After removing the leaf debris, the extracts (50 μL each)

were loaded on a Whatman No. 1 filter paper strip. The samples were chromatographed in a solvent system (150 mM Tris-HCl, pH 7.6/ethanol/1-butanol, v/v, 6/10/1) till the solvent front reached ~1 cm from the top of the paper strip. The filter paper was then wrapped with plastic waterproofing membrane and exposed to X-ray film. Empty circles: the starting line; red arrow: the ^{32}P-NAD^{+} band; black arrows: ^{32}P-NAD^{+} metabolites.

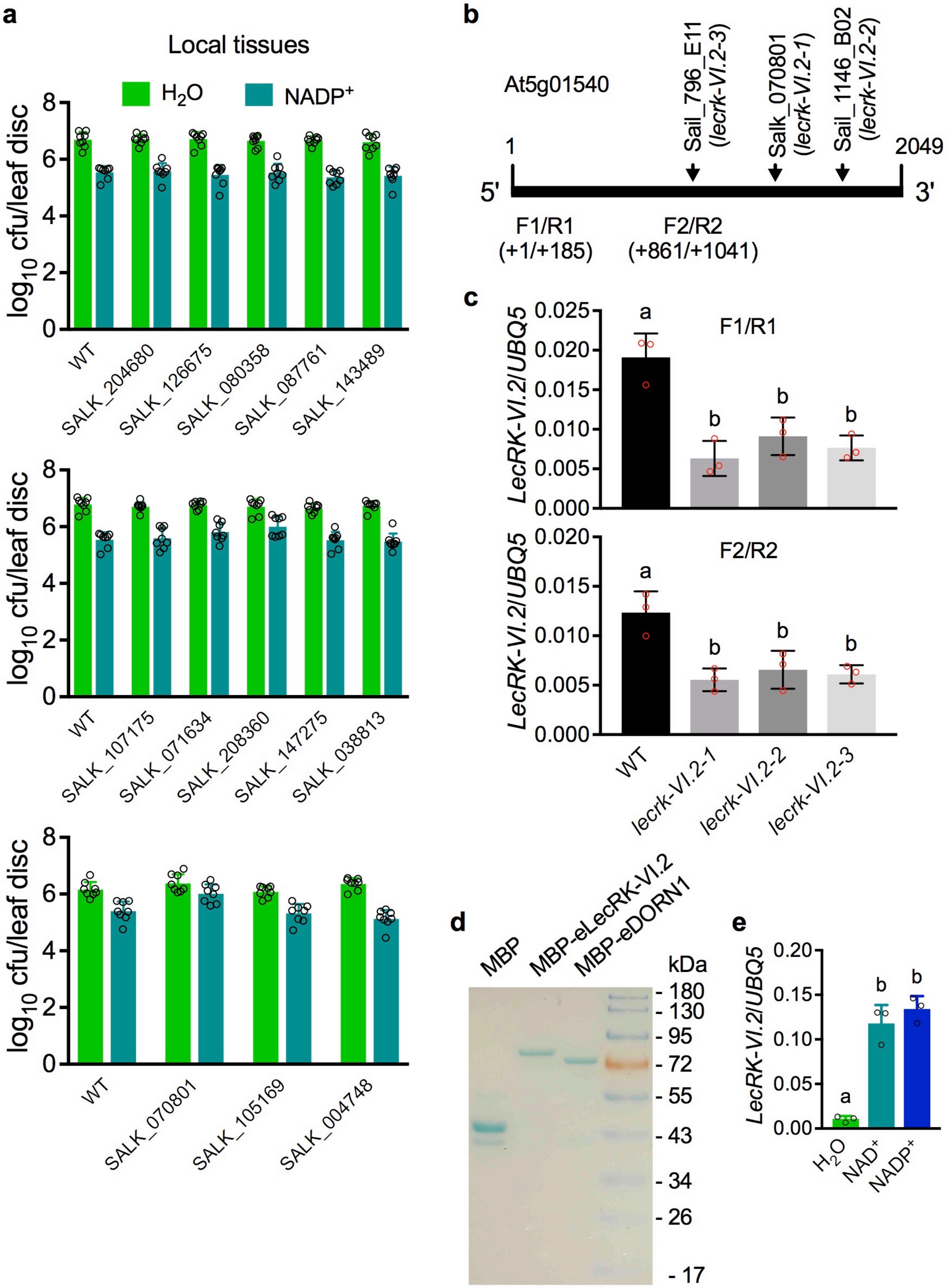

Supplementary Figure 3. Exogenous $NADP^+$-induced local resistance in the previously isolated T-DNA insertion lines and the transcript levels of *LecRK-VI.2* in three T-DNA insertion lines

(**a**) Exogenous $NADP^+$-induced local resistance in the wild-type (WT) and indicated T-DNA insertion lines. Three leaves on each 4-week-old soil-grown plant were infiltrated with H_2O or 0.4 mM $NADP^+$, and the infiltrated leaves were inoculated with *Psm* (OD_{600} = 0.001) 5 hr after the $NADP^+$ treatment. The bacterial growth was determined 3 d later. Data represent the mean ± SD of eight biological replicates. Cfu: colony-forming units.

(**b**) The T-DNA insertion sites in SALK_070801 (*lecrk-VI.2-1*), SAIL_1146_B02 (*lecrk-VI.2-2*), and SAIL_796_E11 (*lecrk-VI.2-3*) and the positions of the two pairs of primers used for qPCR analysis of *LecRK-VI.2* transcript levels in (c).

(**c**) Transcript levels of *LecRK-I.8* in the wild type, *lecrk-VI.2-1*, *lecrk-VI.2-2*, and *lecrk-VI.2-3*. Total RNA was extracted from leaves of 4-week-old soil-grown plants and subjected to qPCR analysis. Expression levels were normalized against *UBQ5*. Data represent the mean ± SD of three biological replicates. Different letters above the bars denote significant differences ($p < 0.05$; one-way ANOVA with Tukey's test).

(**d**) The purified recombinant MBP, MBP-eLecRK-VI.2, and MBP-eDORN1 proteins used for $NAD(P)^+$ binding assays in Figures 2e-2g.

(**e**) Induction of *LecRK-VI.2* by NAD^+ and $NADP^+$. Leaves of 4-week-old soil-grown wild-type Col-0 plants were infiltrated with H_2O, 0.2 mM NAD^+ or 0.4 mM $NADP^+$. Four hr later, the treated leaves were collected. Total RNA was extracted and subjected to qPCR analysis. Expression levels were normalized against *UBQ5*. Data represent the mean ± SD of three biological replicates. Different letters above the bars denote significant differences ($p < 0.05$; one-way ANOVA with Tukey's test).

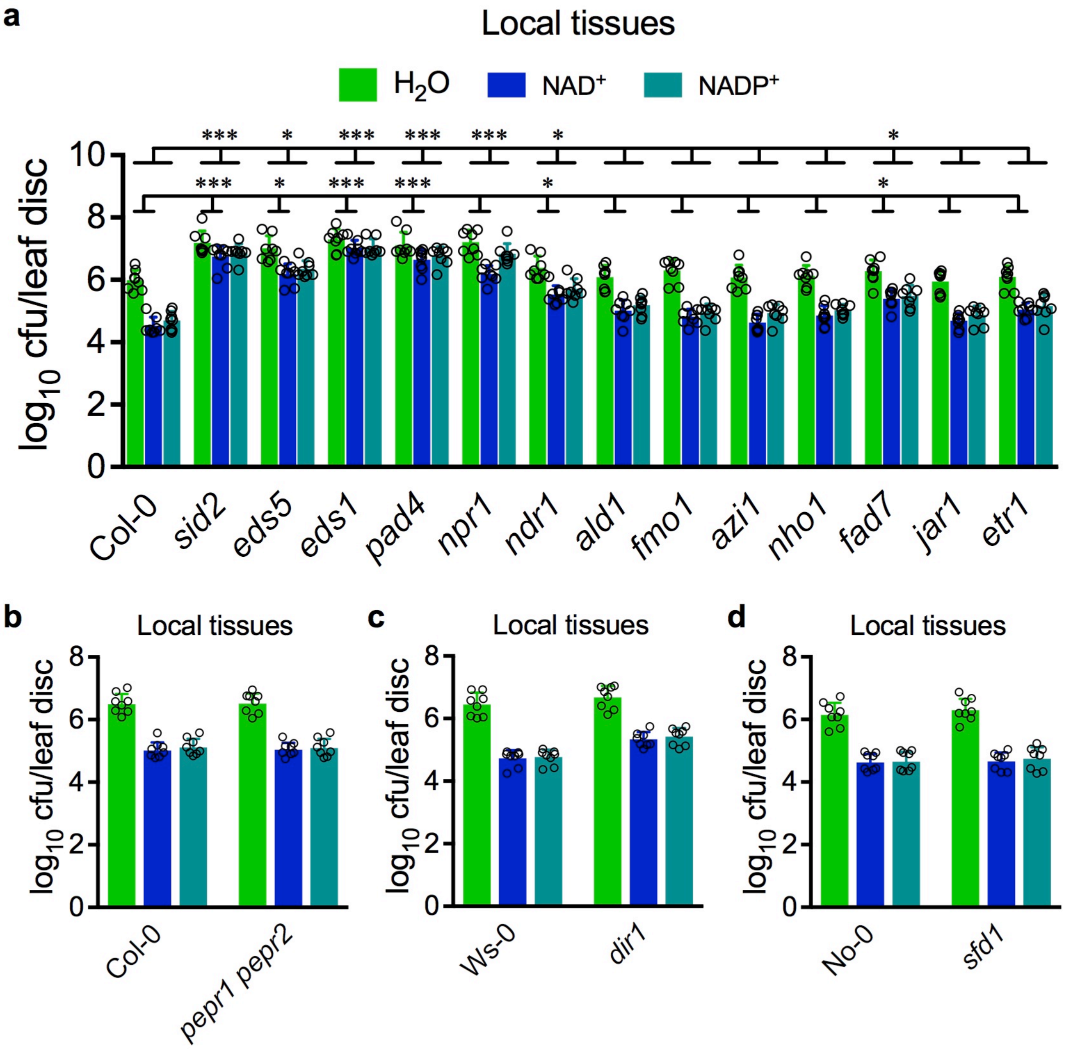

Supplementary Figure 4. Exogenous NAD(P)$^+$-induced local resistance in various SAR mutants

(**a-d**) Exogenous NAD(P)$^+$-induced local resistance in the wild-type Col-0, *sid2*, *eds5*, *eds1*, *pad4*, *npr1*, *ndr1*, *ald1*, *fmo1*, *azi1*, *nho1*, *fad7*, *jar1*, and *etr1* (a), Col-0 and *pepr1 pepr2* (b), the wild-type Ws-0 and *dir1* (c), as well as the wild-type No-0 and *sfd1* (d). Three leaves on each 4-week-old soil-grown plant were infiltrated with H_2O, 0.2 mM NAD$^+$, or 0.4 mM NADP$^+$. The infiltrated leaves were inoculated with *Psm* (OD_{600} = 0.001) 5 hr after the NAD(P)$^+$ treatment, and the bacterial growth was determined 3 d later. Data represent the mean ± SD of eight

biological replicates. Asterisks denote significant differences between the induction in the mutants and that in the wild type (*$p < 0.05$, ***$p < 0.001$; two-way ANOVA with Sidak's test). Upper line: induction by $NADP^+$; lower line: induction by NAD^+.

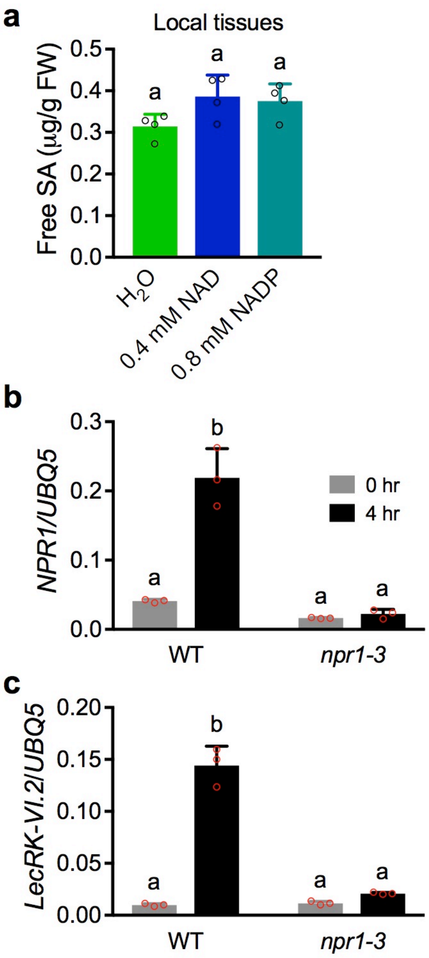

Supplementary Figure 5. $NAD(P)^+$-induced SA accumulation and SA-induced *LecRK-VI.2* expression

(**a**) Leaves of 4-week-old soil-grown wild-type Col-0 plants were infiltrated with H_2O, 0.4 mM NAD^+ or 0.8 mM $NADP^+$. Twenty-four hr later, the treated leaves were collected, and free SA levels in the samples were determined by HPLC. Data represent the mean ± SD of three biological replicates. Same letters above the bars indicate no significant differences ($p > 0.05$;

one-way ANOVA with Tukey's test).

(**b**, **c**) Four-week-old soil-grown wild-type (WT) Col-0 and *npr1-3* mutant plants were sprayed and soil-drenched with 0.5 mM SA water solution. Leaf tissues were collected 4 hr later. Total RNA was extracted and subjected to qPCR analysis. Data represent the mean ± SD of three biological replicates. Different letters above the bars denote significant differences ($p < 0.05$; one-way ANOVA with Tukey's test).

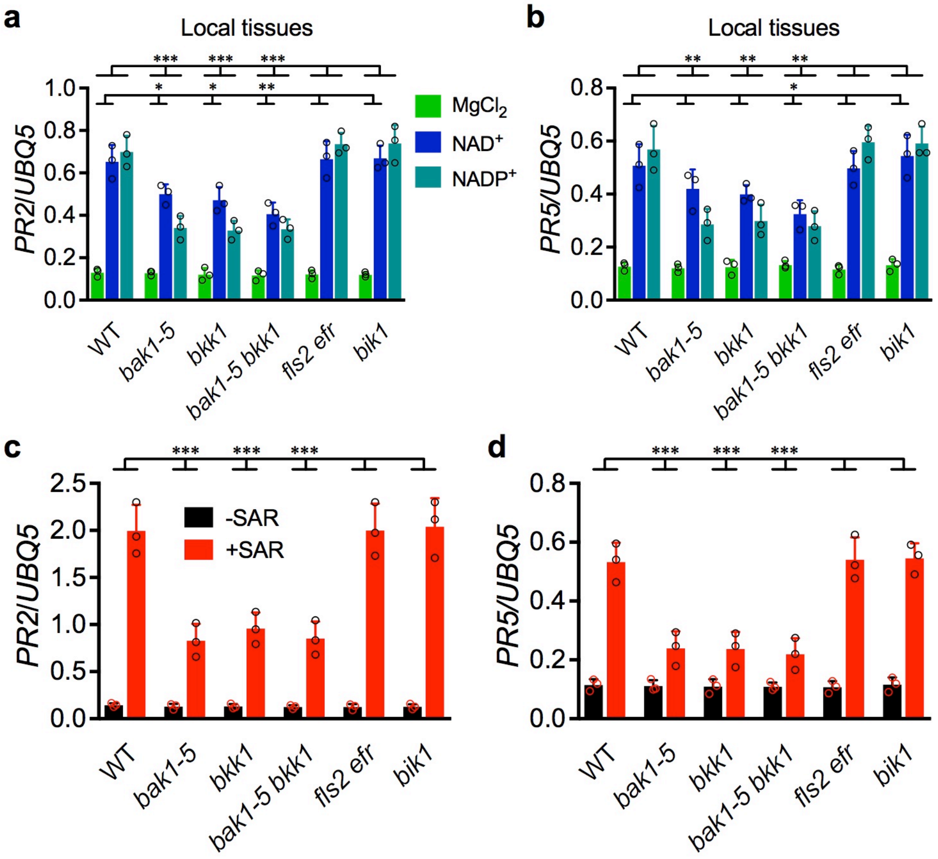

Supplementary Figure 6. *PR2* and *PR5* expression in the local and systemic leaves of several PTI mutants

(**a**, **b**) Exogenous NAD(P)$^+$-induced local expression of *PR2* (a) and *PR5* (b) in the wild-type (WT), *bak1-5*, *bkk1*, *bak1-5 bkk1*, *fls2 efr*, and *bik1* plants. Three leaves on each 4-week-old

soil-grown plant were infiltrated with H_2O, 0.2 mM NAD^+, or 0.4 mM $NADP^+$. The infiltrated leaves were collected 20 hr later for *PR* gene analysis by qPCR. Expression levels were normalized against *UBQ5*. Data represent the mean ± SD of three biological replicates. Asterisks denote significant differences between the induction in the mutants and that in the wild type (**p* < 0.05, ***p* < 0.01, ****p* < 0.001; two-way ANOVA with Sidak's test). Upper line: induction by $NADP^+$; lower line: induction by NAD^+.

(**c**, **d**) Expression of *PR2* (c) and *PR5* (d) in the systemic leaves of the wild-type, *bak1-5*, *bkk1*, *bak1-5 bkk1*, *fls2 efr*, and *bik1* plants with or without SAR induction. Three lower leaves on each 4-week-old soil-grown plant were infiltrated with 10 mM $MgCl_2$ or a *Psm* suspension (OD_{600} = 0.002). Two d later, the systemic leaves were collected for *PR* gene analysis by qPCR. Expression levels were normalized against *UBQ5*. Data represent the mean ± SD of three biological replicates. Asterisks denote significant differences between the induction in the mutants and that in the wild type (****p* < 0.001; two-way ANOVA with Sidak's test).

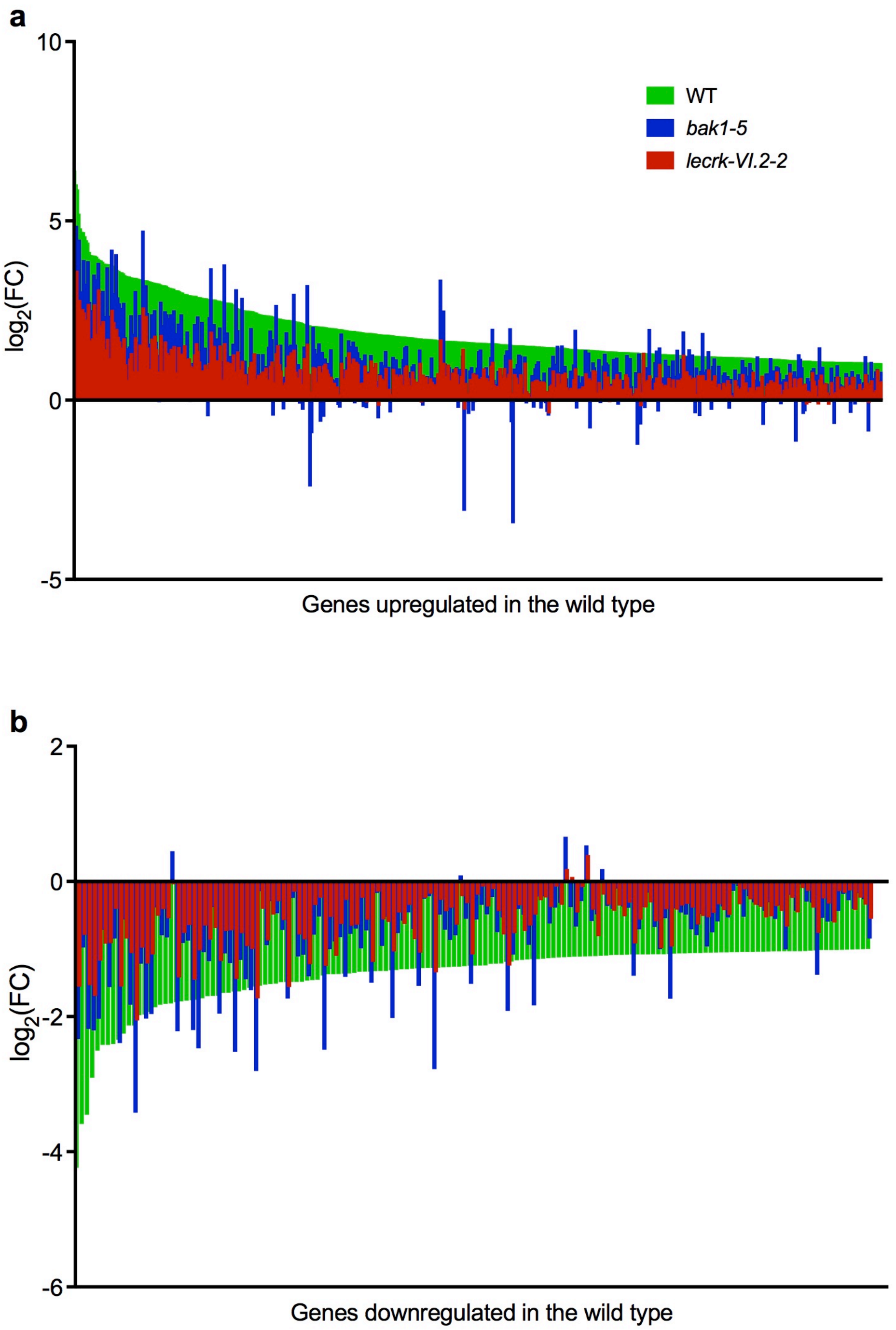

Supplementary Figure 7. Comparison of SAR activation-mediated gene induction and suppression in the wild type, *bak1-5*, and *lecrk-VI.2-2*

(**a**) Induction of the genes, which were induced twofold or higher with a low *q* value (≤ 0.05) in the wild type, in the systemic leaves of the wild type (WT), *bak1-5*, and *lecrk-VI.2-2* after local *Psm* inoculation is presented.

(**b**) Suppression of the genes, which were suppressed twofold or more with a low *q* value (≤ 0.05) in the wild type, in the systemic leaves of the wild type, *bak1-5*, and *lecrk-VI.2-2* after local *Psm* inoculation is presented.

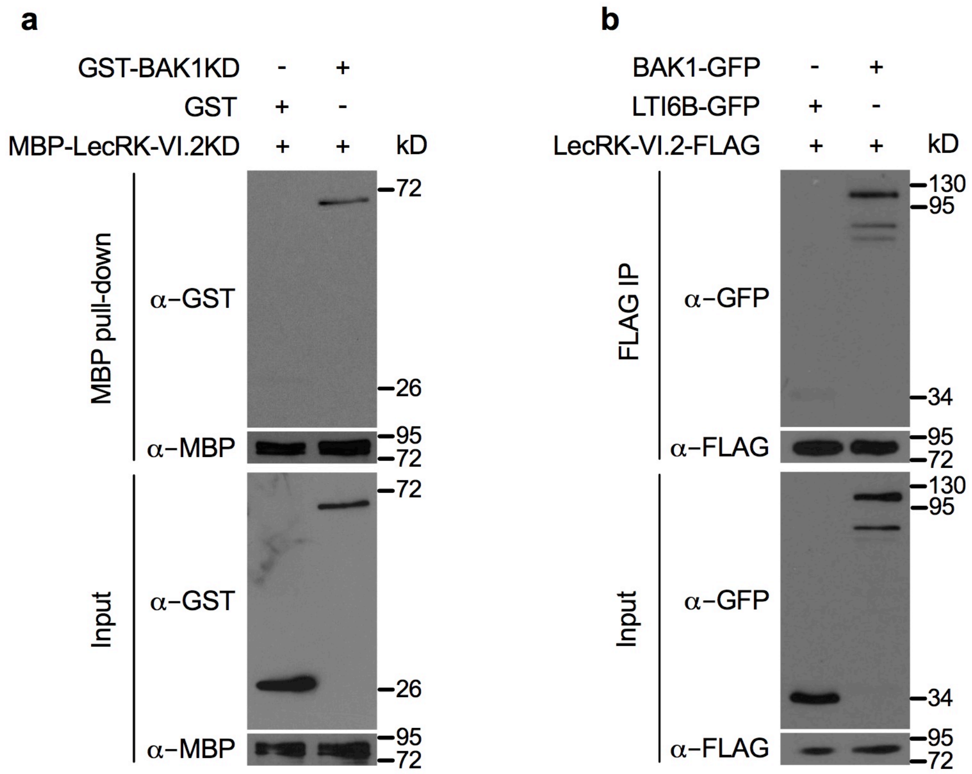

Supplementary Figure 8. LecRK-VI.2 does not interact with GST *in vitro* or LTI6B-GFP in *N. benthamiana*

(**a**) *In vitro* MBP pull-down assay of LecRK-VI.2KD interaction with BAK1KD. Recombinant MBP-LecRK-VI.2KD was incubated with GST-BAK1KD or GST and pulled down with amylose resin beads. Input and bead-bound proteins were analyzed by immunoblotting with

monoclonal anti-GST and anti-MBP antibodies.

(**b**) Co-IP analysis of LecRK-VI.2-FLAG association with BAK1-GFP in *N. benthamiana*. Total proteins (input) of *N. benthamiana* leaves transiently co-expressing LecRK-VI.2-FLAG and BAK1-GFP or LTI6B-GFP were immunoprecipitated with anti-FLAG affinity agarose beads and the precipitates were analyzed by immunoblotting with monoclonal anti-GFP and anti-FLAG antibodies.

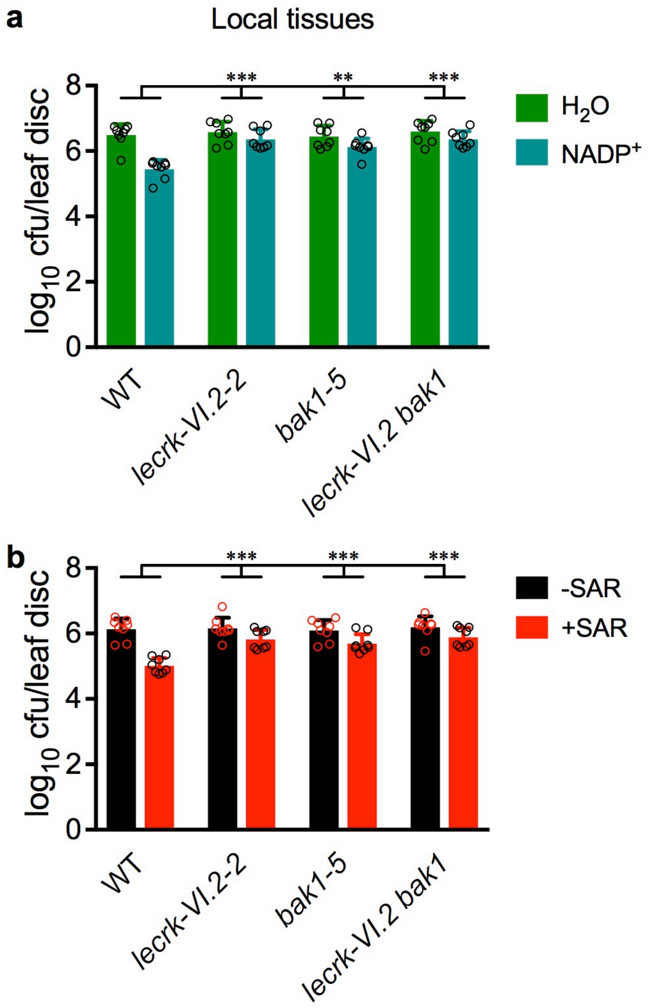

Supplementary Figure 9. Characterization of the *lecrk-VI.2 bak1* double mutant

(**a**) Exogenous $NADP^+$-induced local resistance in the wild-type (WT), *lecrk-VI.2-2*, *bak1-5*, and *lecrk-VI.2 bak1* plants. Leaves of 4-week-old soil-grown plants were infiltrated with 0.4 mM $NADP^+$ or H_2O. Five hr later, the infiltrated leaves were inoculated with a *Psm* suspension ($OD_{600} = 0.001$). Three d later, eight leaves were collected to examine the growth of the pathogen. Data represent the mean ± SD of eight biological replicates. Asterisks denote significant differences between the induction in the mutants and that in the wild type (**$p < 0.01$, ***$p < 0.001$; two-way ANOVA with Sidak's test).

(**b**) Biological induction of SAR in the wild-type, *lecrk-VI.2-2*, *bak1-5*, and *lecrk-VI.2 bak1* plants. Three lower leaves on each 4-week-old soil-grown plant were infiltrated with 10 mM $MgCl_2$ or a *Psm* suspension ($OD_{600} = 0.002$). Two d later, two systemic leaves were challenge-inoculated with *Psm* ($OD_{600} = 0.001$). Three d later, eight leaves were collected to examine the growth of the pathogen. Data represent the mean ± SD of eight biological replicates. Asterisks denote significant differences between the induction in the mutants and that in the wild type (***$p < 0.001$; two-way ANOVA with Sidak's test).

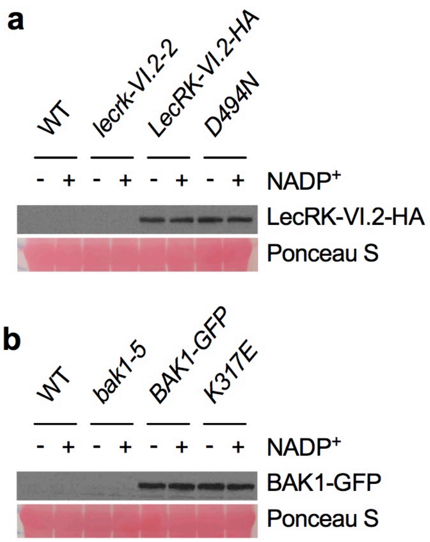

Supplementary Figure 10. Protein levels in the transgenic lines used for characterization in Fig. 7

(**a**) LecRK-VI.2-HA levels in the wild-type (WT), *lecrk-VI.2-2*, *LecRK-VI.2:LecRK-VI.2-HA*, and *LecRK-VI.2:lecrk-VI.2(D494N)-HA* plants with (+) or without (-) 0.8 mM $NADP^+$ for 4 h. Total protein was extracted, subjected to SDS-PAGE, and analyzed by immunoblot using a monoclonal anti-HA antibody. Ponceau S staining of RuBisCO was used as the loading control. (**b**) BAK-GFP levels in the wild-type, *bak1-5*, *35S:BAK1-GFP*, and *35S:bak1(K317)-GFP* plants with (+) or without (-) 0.8 mM $NADP^+$ for 4 h. Total protein was extracted, subjected to SDS-PAGE, and analyzed by immunoblot using a polyclonal anti-GFP antibody. Ponceau S staining of RuBisCO was used as the loading control.

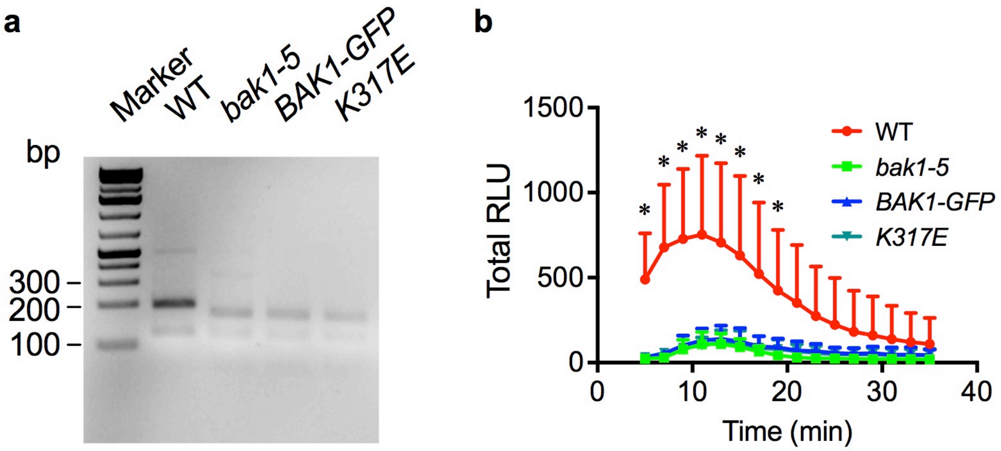

Supplementary Figure 11. BAK1-GFP is unable to complement *bak1-5* in flg22-induced ROS burst

(**a**) Confirmation of the *bak1-5* genetic background of the *35S:BAK1-GFP/bak1-5* (*BAK1-GFP*) and *35S:bak1(K317E)-GFP/bak1-5* (*K317E*) transgenic plants. To avoid interference of the transgene, the genomic DNA region carrying the *bak1-5* mutation was first amplified by PCR using an intron primer and the PCR products were then analyzed with the *bak1-5* dCAPS marker. (**b**) ROS production triggered by 100 nM flg22 in the wild-type (WT), *bak1-5*, *35S:BAK1-GFP/bak1-5*, and *35S:bak1(K317E)-GFP/bak1-5* leaf disks measured in a luminol-based assay as relative light units (RLUs). Data represent the mean ± SD of 12 biological replicates. Asterisks denote significant differences between the wild type and other genotypes (*$p < 0.05$; Student's t test).

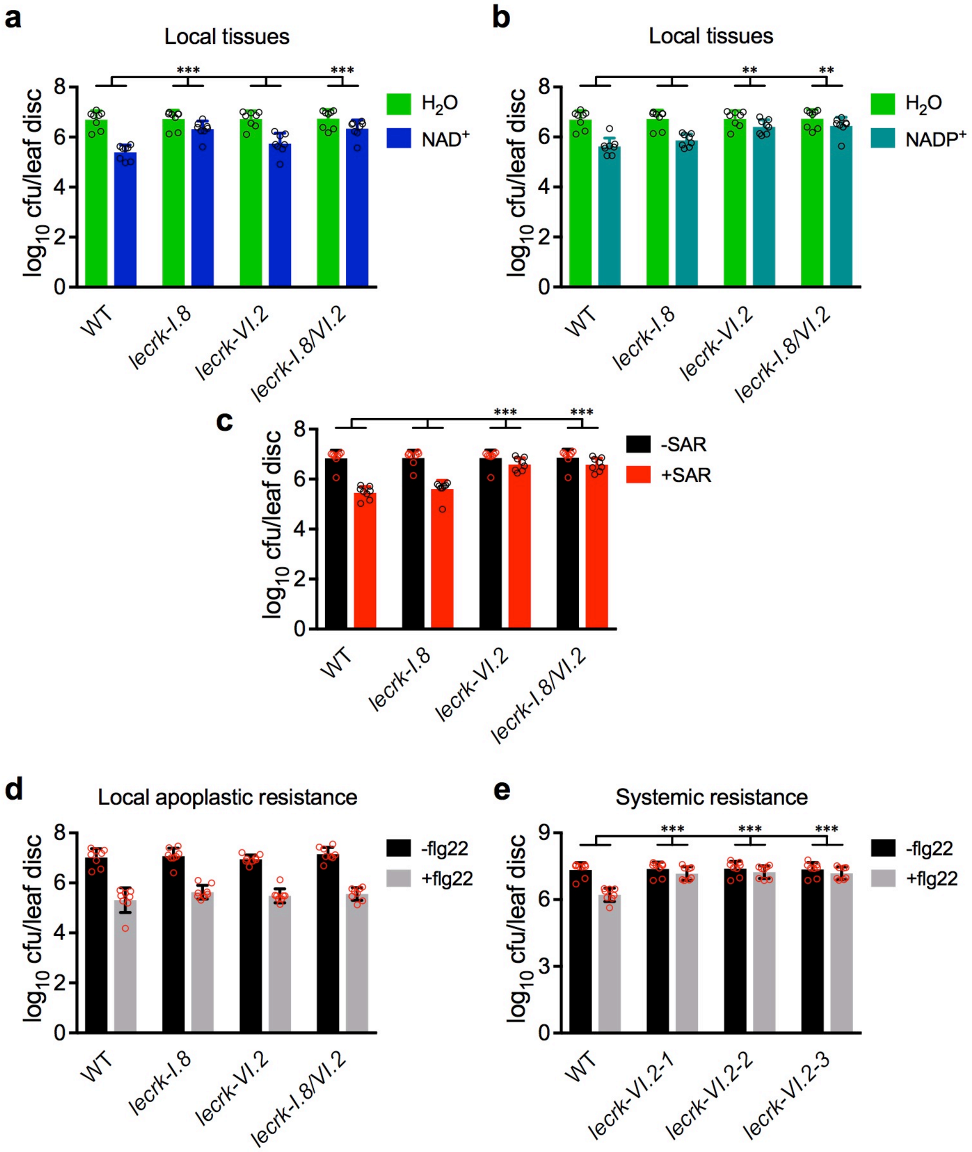

Supplementary Figure 12. Characterization of the *lecrk-I.8/VI.2* double mutant

(**a**, **b**) Exogenous NAD^+ (a) and $NADP^+$ (b) -induced local resistance in the wild-type (WT), *lecrk-I.8*, *lecrk-VI.2*, and *lecrk-I.8/VI.2* plants. Leaves of 4-week-old soil-grown plants were infiltrated with 0.2 mM NAD^+, 0.4 mM $NADP^+$ or H_2O. Five hr later, the infiltrated leaves were inoculated with a *Psm* suspension (OD_{600} = 0.001). Three d later, eight leaves were collected to examine the growth of the pathogen. Data represent the mean ± SD of eight biological

replicates. Asterisks denote significant differences between the induction in the mutants and that in the wild type (**$p < 0.01$, ***$p < 0.001$; two-way ANOVA with Sidak's test).

(**c**) Biological induction of SAR in the wild-type, *lecrk-I.8*, *lecrk-VI.2*, and *lecrk-I.8/VI.2* plants. Three lower leaves on each 4-week-old soil-grown plant were infiltrated with 10 mM $MgCl_2$ or a *Psm* suspension ($OD_{600} = 0.002$). Two d later, two systemic leaves were challenge-inoculated with *Psm* ($OD_{600} = 0.001$). Three d later, eight leaves were collected to examine the growth of the pathogen. Data represent the mean ± SD of eight biological replicates. Asterisks denote significant differences between the induction in the mutants and that in the wild type (***$p < 0.001$; two-way ANOVA with Sidak's test).

(**d**) Flg22-induced local resistance in the wild-type, *lecrk-I.8*, *lecrk-VI.2*, and *lecrk-I.8/VI.2* plants. Leaves of 4-week-old soil-grown plants were infiltrated with 100 nM flg22 or H_2O. Twenty-four hr later, the infiltrated leaves were inoculated with a *Psm* suspension ($OD_{600} = 0.001$). Three d later, eight leaves were collected to examine the growth of the pathogen. Data represent the mean ± SD of eight biological replicates.

(**e**) Flg22-induced systemic resistance in the wild-type and *lecrk-VI.2* mutant plants. Three lower leaves on each 4-week-old soil-grown plant were infiltrated with H_2O or 200 nM flg22 every 12 hr for a total of 4 times. About 5 hr after the last infiltration, two systemic leaves were challenge-inoculated with a *Psm* suspension ($OD_{600} = 0.001$). Three d later, eight leaves were collected to examine the growth of the pathogen. Data represent the mean ± SD of eight biological replicates. Asterisks denote significant differences between the induction in the mutants and that in the wild type (***$p < 0.001$; two-way ANOVA with Sidak's test).

Supplementary Table S1. Primers used in this study

Primer name	Sequence (5' to 3')
XbaI-eLecRK-VI.2F	GCTCTAGACATGTTCGAGCTCAGAGAAC
HindIII-eLecRK-VI.2R	CCCAAGCTTCAGACTTGAGAATTTAAACCTTCCTTC
BglII-LecRK-VI.2KDF	GAAGATCTTTCTTCGTCATGTACAAGAAGAG
XhoI-LecRK-VI.2KDR	CCCTCGAGCTACTGACTGATACGAGAAGTC
EcoRI-BAK1KDF	GGAATTCCGAAGGAAAAAGCCGCAGGAC
SalI-BAK1KDR	GCGTCGACTTATCTTGGACCCGAGGGGTA
XbaI-LecRK-VI.2F	GCTCTAGATGGGCACACAAAGATCCATG
XhoI-LecRK-VI.2-noStopR	CCCTCGAGCTGACTGATACGAGAAGTCGAAGAAAC
SacI-BAK1F	CGAGCTCATGGAACGAAGATTAATGATCCCTTG
SalI-BAK1-noStopR	GCGTCGACTCTTGGACCCGAGGGGTATTCGTTTTCG
XbaI-LecRK-VI.2PF	GCTCTAGACGGAGATGATGATGTACCTG
BglII-3×HA-LecRK-VI.2R	GAAGATCTCACGCATAGTCAGGAACATCGTATGGGTACGCATAGTC AGGAACATCGTATGGGTACGCATAGTCAGGAACATCGTATGGGTAC TGACTGATACGAGAAGTCGAAGAAAC
LecRK-VI.2(D494N)F	GAGAAGATTGTGATTCATAGAAACGTGAAACCTAGCAATGTC
LecRK-VI.2(D494N)R	GACATTGCTAGGTTTCACGTTTCTATGAATCACAATCTTCTC
BAK1(K317E)F	GGTACTTTAGTGGCCGTTGAAAGGCTAAAAGAGGAGCG
BAK1(K317E)R	CGCTCCTCTTTTAGCCTTTCAACGGCCACTAAAGTACC
KpnI-LTI6BF	GGGGTACCATGAGTACAGCCACTTTCGTAG
XbaI-LTI6BR	GCTCTAGACTTGGTGATGATATAAAGAGCG
BAK1intronF	AACTTGCTTTACCTGTTTCCTC
BAK1intronR	TAACACAAATAGAGGTAACACAG
bak1-5dCAPSF	AAGAGGGCTTGCGTATTTACATGATCAGT
bak1-5dCAPSR	GAGGCGAGCAAGATCAAAAG
qLecRK-VI.2F1	ATGGGCACACAAAGATCCATG
qLecRK-VI.2R1	GTGAGTCTCAATAGTCCGTC
qLecRK-VI.2F2	TCTAATCCTCTCGGAGCTTC
qLecRK-VI.2R2	CCAGTCTTCAAGAGTCTCTTC
qPR1F	CTCATACACTCTGGTGGG
qPR1R	ATTGCACGTGTTCGCAGC
qPR2F	ATCAAGGAGCTTAGCCTCAC
qPR2R	TGTAAAGAGCCACAACGTCC
qPR5F	CTCTTCCTCGTGTTCATCAC
qPR5R	GAAGCACCTGGAGTCAATTC
qUBQ5F	TCTCCGTGGTGGTGCTAAG
qUBQ5R	GAACCTTTCCAGATCCATCG

Supplementary Methods

SA and ROS measurement

Free SA levels and ROS burst were measured as previously reported [1, 2]. For free SA assay, 0.1 g tissues were ground in liquid nitrogen and extracted with 1 mL of 90% methanol. After

centrifugation at 14,000 ×*g* for 10 min, the supernatant was transferred into a microcentrifuge tube. The pellet was extracted with 0.5 mL of 100% methanol and the supernatant was transferred to the same tube and dried in a speed vacuum to final volume of ~50 μL. The residue was resuspended to 0.5 mL of 0.1 M sodium acetate buffer, pH 5.5. An equal volume of 10% trichloroacetic acid was added to the tube. After centrifugation at 14,000 ×*g* for 10 min, the supernatant was transferred to a fresh tube and partitioned with 1 mL extraction solvent (1 ethylacetate : 1 cyclohexane). The top organic phase was transferred to a new tube, and dried in a speed vacuum to final volume ~25 μL. The residue was resuspended to 0.25 mL with 0.2 M sodium acetate buffer (pH 5.5). After centrifugation at 14,000 ×*g* for 10 min, the supernatant was used for HPLC analysis. The sample was eluted with 0.2 M sodium acetate buffer pH 5.5 in 10% methanol at a flow-rate of 0.80 mL min^{-1}. For ROS burst assay, leaf disks (~6 mm in diameter) were collected from newly expanded leaves and each disc was placed in a well of a black 96-well plate containing 150 μL of water. The plate was covered with a transparent lid and kept at room temperature overnight. After removing water, 100 μL of assay solution (100 μM luminal, 10 μg mL^{-1} peroxidase, and 100 nM flg22) was added into each well, and light emission was immediately measured using a Veritas™ Microplate Luminometer (Promega Corporation, Sunnyvale, CA).

Paper Chromatography

Paper chromatography was conducted as previously described [3]. Briefly, leaves were submerged in 1 mL 80% ethanol and boiled at 95°C for 2 min. After removing the leaf debris, the extracts (50 μL each) were loaded on a Whatman No. 1 filter paper strip. The samples were chromatographed in a solvent system (150 mM Tris-HCl, pH 7.6/ethanol/1-butanol, v/v, 6/10/1)

till the solvent front reached ~1 cm from the top of the paper strip. The filter paper was then wrapped with plastic waterproofing membrane and exposed to X-ray film.

Supplementary References

1. Marek, G. et al. A high-throughput method for isolation of salicylic acid metabolic mutants. Plant Methods 6, 21 (2010).
2. Roux, M. et al. The *Arabidopsis* leucine-rich repeat receptor-like kinases BAK1/SERK3 and BKK1/SERK4 are required for innate immunity to hemibiotrophic and biotrophic pathogens. Plant Cell 23, 2440-2455 (2011).
3. Chan, P. C. & Bielski, B. H. Lactate dehydrogenase-catalyzed stereospecific hydrogen atom transfer from reduced nicotinamide adenine dinucleotide to dicarboxylate radicals. J Biol Chem 250, 7266-7271 (1975).
