# Supplementary material for: Extracellular pyridine nucleotides trigger plant systemic immunity through a lectin receptor kinase/BAK1 complex
Source: Nat Commun. 2019 Oct 22;10:4810. doi: 10.1038/s41467-019-12781-7 (PMC6805918; doi:10.1038/s41467-019-12781-7)
Supplement: Supplementary file 2 — Description of Additional Supplementary Files [file 41467_2019_12781_MOESM2_ESM.pdf]

Description of Additional Supplementary Files

File Name: Supplementary Data 1

Description: SAR activation-induced transcriptome changes in the wild type, lecrk-VI.2-2, and bak1-5. The data were sorted by the log2(FC) values in the wild type (WT) (column D), log2(FC) values ≥ 1 and ≤ 1 are highlighted in red and blue, respectively, and q values ≤ 0.05 are highlighted in red. FC: fold change.
