# Supplementary material for: Extracellular pyridine nucleotides trigger plant systemic immunity through a lectin receptor kinase/BAK1 complex
Source: Nat Commun. 2019 Oct 22;10:4810. doi: 10.1038/s41467-019-12781-7 (PMC6805918; doi:10.1038/s41467-019-12781-7)
Supplement: Supplementary file 4 — Reporting Summary [file 41467_2019_12781_MOESM4_ESM.pdf]

nature research

Corresponding author(s): Zhonglin Mou

Last updated by author(s): Sep 25, 2019

Reporting Summary

Nature Research wishes to improve the reproducibility of the work that we publish. This form provides structure for consistency and transparency in reporting. For further information on Nature Research policies, see Authors & Referees and the Editorial Policy Checklist .

Statistics

For all statistical analyses, confirm that the following items are present in the figure legend, table legend, main text, or Methods section.

n/a	Confirmed	
☐	☒	The exact sample size (*n*) for each experimental group/condition, given as a discrete number and unit of measurement
☐	☒	A statement on whether measurements were taken from distinct samples or whether the same sample was measured repeatedly
☒	☐	The statistical test(s) used AND whether they are one- or two-sided<br>*Only common tests should be described solely by name; describe more complex techniques in the Methods section.*
☒	☐	A description of all covariates tested
☒	☐	A description of any assumptions or corrections, such as tests of normality and adjustment for multiple comparisons
☒	☐	A full description of the statistical parameters including central tendency (e.g. means) or other basic estimates (e.g. regression coefficient) AND variation (e.g. standard deviation) or associated estimates of uncertainty (e.g. confidence intervals)
☒	☐	For null hypothesis testing, the test statistic (e.g. *F*, *t*, *r*) with confidence intervals, effect sizes, degrees of freedom and *P* value noted<br>*Give P values as exact values whenever suitable.*
☒	☐	For Bayesian analysis, information on the choice of priors and Markov chain Monte Carlo settings
☒	☐	For hierarchical and complex designs, identification of the appropriate level for tests and full reporting of outcomes
☐	☒	Estimates of effect sizes (e.g. Cohen's *d*, Pearson's *r*), indicating how they were calculated

Our web collection on statistics for biologists contains articles on many of the points above.

Software and code

Policy information about availability of computer code

Data collection	Affymetrix Expression Console Software (version 3.0) was used for microarray probe set summarization and initial data quality examination.
Data analysis	R Software (version 3.4.2), Affy software (version 1.58), and Limma software (version3.36) were used for microarray data analysis. Microsoft Excel of Microsoft Office 2011 for Macintosh was used for Student's t test, and Prism 7 was used for one-way and two-way ANOVA analyses.

For manuscripts utilizing custom algorithms or software that are central to the research but not yet described in published literature, software must be made available to editors/reviewers. We strongly encourage code deposition in a community repository (e.g. GitHub). See the Nature Research guidelines for submitting code & software for further information.

Data

Policy information about availability of data

All manuscripts must include a data availability statement. This statement should provide the following information, where applicable:

- Accession codes, unique identifiers, or web links for publicly available datasets
- A list of figures that have associated raw data
- A description of any restrictions on data availability

The microarray data that support the findings of this study have been deposited in the Gene Expression Omnibus database with the accession number GSE121886.

Field-specific reporting

Please select the one below that is the best fit for your research. If you are not sure, read the appropriate sections before making your selection.

Life sciences study design

All studies must disclose on these points even when the disclosure is negative.

Sample size	For RNA analysis, three independent biological samples were analyzed, and each sample was collected from 12 plants. For bacterial pathogen growth assessment, three leaves on each plant were inoculated and a total of 12 plants were used for each genotype/treatment. Eight leaves per genotype/treatment were collected to examine the growth of the pathogen. A leaf disk was collected from each leaf and used as a independent biological sample.
Data exclusions	No data were excluded from the analyses.
Replication	All experiments were repeated at least three times with similar results.
Randomization	For RNA sample collection, one leaf from each of the 12 plants was collected, cut into small pieces, and mixed, then 100 mg leaf tissues were taken from the mixture for RNA extraction. For pathogen growth assessment, eight leaves were randomly taken from a total of 36 leaves (3 x 12).
Blinding	Blinding was not possible for the described experiments, as the investigator had to carefully label each genotype/treatment to ensure that samples were not messed up.

Reporting for specific materials, systems and methods

We require information from authors about some types of materials, experimental systems and methods used in many studies. Here, indicate whether each material, system or method listed is relevant to your study. If you are not sure if a list item applies to your research, read the appropriate section before selecting a response.

Materials & experimental systems

n/a	Involved in the study	
☐	☒	Antibodies
☒	☐	Eukaryotic cell lines
☒	☐	Palaeontology
☒	☐	Animals and other organisms
☒	☐	Human research participants
☒	☐	Clinical data

Methods

n/a	Involved in the study	
☒	☐	ChIP-seq
☒	☐	Flow cytometry
☒	☐	MRI-based neuroimaging

Antibodies

Antibodies used	anti-MBP, NEB, cat# E8032S; anti-GST, Cell Signaling, cat# 2625S; anti-FLAG, Sigma, cat# F1804; polyclonal and monoclonal anti-GFP, monoclonal anti-HA, Santa Cruz Biotechnology, cat# sc-8334, sc-9996, and sc-7392, respectively.
Validation	All antibodies were tested for specificities by detecting tagged proteins. All antibodies recognize tagged proteins, though unspecific bands were also detected.
